# A Single RNaseIII Domain Protein from *Entamoeba histolytica* Has dsRNA Cleavage Activity and Can Help Mediate RNAi Gene Silencing in a Heterologous System

**DOI:** 10.1371/journal.pone.0133740

**Published:** 2015-07-31

**Authors:** Justine M. Pompey, Bardees Foda, Upinder Singh

**Affiliations:** 1 Department of Microbiology and Immunology, Stanford University School of Medicine, Stanford, California, United States of America; 2 Division of Infectious Diseases, Department of Internal Medicine, Stanford University School of Medicine, Stanford, California, United States of America; 3 Molecular Genetics and Enzymology Department, National Research Centre, Dokki, Egypt; Wuhan University, CHINA

## Abstract

Dicer enzymes process double-stranded RNA (dsRNA) into small RNAs that target gene silencing through the RNA interference (RNAi) pathway. Dicer enzymes are complex, multi-domain RNaseIII proteins, however structural minimalism of this protein has recently emerged in parasitic and fungal systems. The most minimal Dicer, *Saccharomyces castellii* Dicer1, has a single RNaseIII domain and two double stranded RNA binding domains. In the protozoan parasite *Entamoeba histolytica* 27nt small RNAs are abundant and mediate silencing, yet no canonical Dicer enzyme has been identified. Although EhRNaseIII does not exhibit robust dsRNA cleavage *in vitro*, it can process dsRNA in the RNAi-negative background of *Saccharomyces cerevisiae*, and in conjunction with *S*. *castellii* Argonaute1 can partially reconstitute the RNAi pathway. Thus, although EhRNaseIII lacks the domain architecture of canonical or minimal Dicer enzymes, it has dsRNA processing activity that contributes to gene silencing via RNAi. Our data advance the understanding of small RNA biogenesis in *Entamoeba* as well as broaden the spectrum of non-canonical Dicer enzymes that contribute to the RNAi pathway.

## Introduction

Small RNAs (sRNAs) and the RNA interference (RNAi) pathway play critical roles in diverse processes across many eukaryotic systems [1–5]. Since the discovery that small non-coding RNAs can direct targeted gene silencing, multiple classes of sRNAs and sRNA biogenesis pathways have been identified [6, 7]. In the classical primary RNAi pathway, Dicer (an RNaseIII endonuclease) recognizes and cleaves double-stranded RNA (dsRNA) into 20-30bp fragments that have 5'-monophosphate termini [6]. These duplexes are loaded into an RNA-Induced Silencing Complex (RISC) where one strand is preferentially retained to guide sequence-specific silencing of its target mRNA by Argonaute (Ago), a key component of RISC [8]. In nematodes, an amplified silencing response occurs through the production of secondary sRNAs by RNA-dependent RNA Polymerase (RdRP) [9–11]. These secondary sRNAs are characterized by 5'-polyphosphate (5'-polyP) termini and are loaded into secondary AGOs to mediate silencing [12]. The only system other than nematodes in which 5'-polyP secondary sRNAs have been described is the parasitic protist *Entamoeba histolytica* [13]. In *E*. *histolytica*, 5'-polyP sRNAs mediate nuclear RNAi and highly stable transcriptional gene silencing of virulence genes [14, 15].

Although conserved in function, the structure of Dicer enzymes varies among eukaryotes [8]. Canonical Dicer enzymes contain a PAZ domain, which binds the 3'-end of the sRNA, and two RNaseIII domains, which dimerize to form the catalytic center of the enzyme [16, 17]. Some canonical Dicer enzymes have additional domains such as an N-terminal helicase domain, a domain of unknown function (DUF 283), or a double-stranded RNA binding domain [dsRBD) [16]. However, not all Dicer enzymes exhibit this canonical structure. Both *Trypanosoma brucei* Dicers contain two RNaseIII domains but lack any other recognizable domains [18, 19]. The most minimal eukaryotic Dicers are found in budding yeast, which contain a single RNaseIII domain and two C-terminal dsRBDs, although only one dsRBD is required for full activity *in vitro* [20, 21]. As RNA hydrolysis requires dimerization of two RNaseIII domains, budding yeast Dicers form homodimers to function [21]. Although Dicer enzymes play key roles in many RNAi pathways, Dicer-independent biogenesis of sRNAs also occurs. These include microRNA-451, for which AGO2 mediates cleavage to the mature species, and Piwi-interacting small RNAs [6, 22, 23].

*Entamoeba histolytica* is an important human pathogen and the second leading cause of parasitic death worldwide [24]. A complex repertoire of sRNAs has been identified in this protozoan parasite including abundant 27nt sRNAs in the trophozoite stage [13]. The 27nt heterogenous sRNA population has 5'-polyP termini, associates with the abundant and nuclear localized EhAgo2-2, and mediates stable transcriptional gene silencing (13, 14]. The 5'-polyP structure of amebic sRNAs is similar to that of secondary sRNAs in nematodes, indicating that they are generated in a Dicer-independent manner [13, 16]. However, even in systems with secondary RNAi pathways, the process is initiated by a primary small interfering RNA (siRNA), which is generated by Dicer-dependent cleavage [25]. Thus, the amplified silencing pathway, mediated by 5'-polyP sRNAs, also requires Dicer processing to initiate the cascade. The *E*. *histolytica* genome encodes several core elements of the RNAi machinery including three Ago proteins (EhAgo2-1, EhAgo2-2, and EhAgo2-3) and two RdRP enzymes (EhRdRP1 and EhRdRP2). Despite a robust endogenous RNAi pathway, a canonical Dicer enzyme is notably absent in *E*. *histolytica* leaving open the question of how the secondary RNAi pathway is initiated and whether further divergence of the Dicer enzyme may have occurred in this parasitic protist. A gene with a single RNaseIII-domain (EHI_068740) is encoded in the genome [26]. This gene has been shown to have some dsRNA cleavage activity, although the specific size of the cleavage product and its loading into the RNAi pathway have not been demonstrated [27].

In this study we explore whether EhRNaseIII (EHI_068740), the *E*. *histolytica* protein with a single RNaseIII domain but without a dsRBD or other domains typically found in Dicer enzymes, can function to contribute to gene silencing via RNAi. We found that while unable to robustly cleave dsRNA *in vitro* under standard experimental conditions, EhRNaseIII is capable of processing dsRNA and contributing to gene silencing in a heterologous system, *Saccharomyces cerevisiae*. Our results not only broaden the understanding about potential sRNA biogenesis mechanisms in *E*. *histolytica* but also potentially expand the repertoire of non-canonical proteins and their contributions to RNAi pathways in non-model systems.

## Results

### The *E*. *histolytica* genome contains a single gene (EHI_068740) with an RNaseIII domain

**Fig 1:**
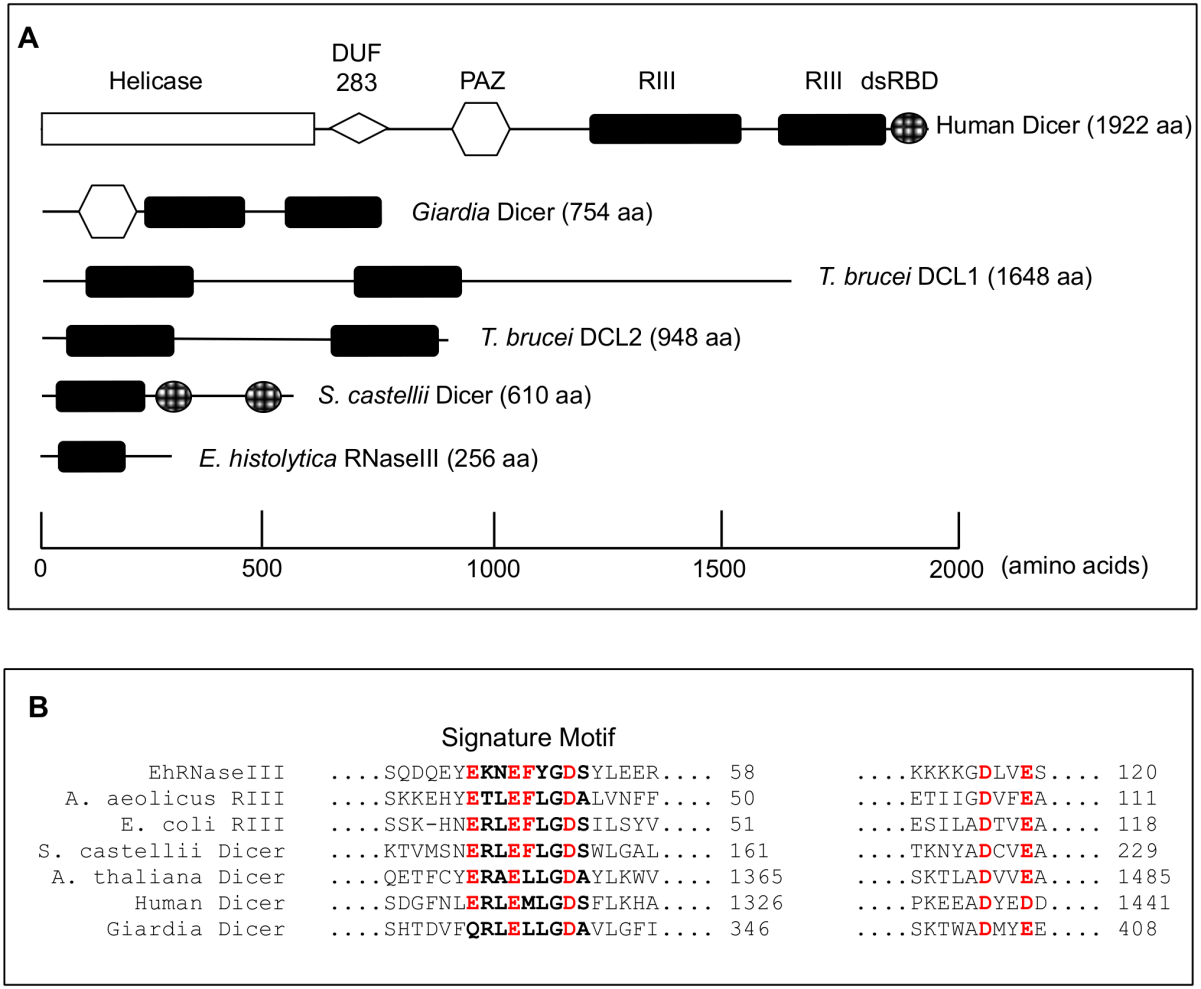
Comparison of RNaseIII proteins. **(A)** Schematic of domain structure of Dicer enzymes across organisms including Human Dicer (Accession Q9UPY3), *Giardia lamblia* Dicer (Accession EDO77862), *Trypanosoma brucei* Dicer 1 (Accession Tb927.8.2370), *Trypanosoma brucei* Dicer 2 (Accession Tb927.3.1230), *Saccharomyces castellii* Dicer 1 (Accession DAA12515), and *E*. *histolytica* RNaseIII (EHI_068740). Scale along the bottom indicates the approximate location of each domain. **(B)** ClustalW alignment of RNaseIII domains across organisms including EhRNaseIII, *Escherichia coli* RNaseIII (Accession AIL18413), *Aquifex aeolicus* RNaseIII (Accession NP_213645), *S*. *castellii* Dicer1, *Arabidopsis thaliana* Dicer (Accession AEZ02177), Human Dicer, and Giardia Dicer. Full-length sequences aligned using Geneious version R7 (Biomatters Ltd) [31]. Excerpt of alignment of catalytic residues shown. RNaseIII signature motif is shown in bold. Important catalytic residues are shown in red. Full-length alignment of EhRNaseIII is available in S1 Fig.

A canonical Dicer enzyme that contains two RNaseIII domains and a PAZ or dsRBD domains has not been identified in the *E*. *histolytica* genome [28]. Instead, only a single gene with a single RNaseIII domain and no other conserved domains is annotated in the genome (Fig 1A). In order to identify other potential Dicer candidate genes, we conducted a bioinformatics search of the *E*. *histolytica* genome for the most common domains found in Dicer enzymes (RNaseIII and PAZ) using profile Hidden Markov Models (HMM). For each domain we retrieved the Pfam-generated HMM (http://pfam.sanger.ac.uk) as well as built our own HMM from a multiple sequence alignment of RNaseIII- or PAZ-containing proteins. To identify domains that may have been mis-annotated or overlooked in the genome annotation, we translated the *E*. *histolytica* genome in six frames and queried both the annotated and translated genomes with each HMM. Hits to each HMM with an e-value ≤ 0.5 were retained as viable candidates. EhRNaseIII was identified by both the Pfam RNaseIII HMM as well as the custom RNaseIII HMM in both the annotated and translated genomes, and was the only hit to meet the significance criteria (S1 Table). The PAZ domain HMM identified the three amebic Argonaute genes but no other genes that met the significance criteria (S1 Table). The possibility that the genome sequencing missed genomic regions with PAZ or RNaseIII domains given the 12.5x coverage is relatively low [28]. Thus, these data indicate that *E*. *histolytica* does not contain a gene with domain features typical of a canonical Dicer enzyme. However, EhRNaseIII does contain all the conserved and catalytic residues characteristic of RNaseIII enzymes (Fig 1B and S1 Fig) [29, 30].

EhRNaseIII (EHI_068740) thus remains the only potential Dicer-like candidate in *E*. *histolytica*. The lack of a PAZ domain suggests that EhRNaseIII may require a cofactor that binds dsRNA in order to function efficiently. *Caenorhabditis elegans* Dicer-1, which fits the canonical Dicer structure, requires a dsRNA binding protein, RDE-4, to cleave dsRNA [32–34]. Alternatively, although no other domains have been identified in EhRNaseIII, it is possible that a divergent dsRBD may be present. Importantly, not all Dicers contain PAZ domains as notably both *T*. *brucei* Dicer enzymes lack identifiable PAZ or dsRBDs and yet are fully functional [18, 19]. Therefore, it remains to be determined whether EhRNaseIII is itself capable of functioning as a Dicer-like enzyme or whether additional proteins are required for activity.

### EhRNaseIII has limited dsRNA cleavage activity in vitro

To determine the role of EhRNaseIII in the amebic RNAi pathway, we attempted to downregulate EhRNaseIII through a variety of techniques including antisense strategies and an RNAi-trigger approach [35]; however, they were not successful [36]. Additionally, we tried to impair endogenous EhRNaseIII protein activity using multiple dominant-negative approaches, which also proved ineffective in changing the abundance of amebic sRNA populations (Pompey and Singh, unpublished data). Given that EhRNaseIII is the sole RNaseIII domain gene in the genome and could be necessary for multiple aspects of RNA processing, we attributed the lack of downregulation as potentially indicating essential cellular function(s). With the difficulties of genetic manipulation, we turned to an *in vitro* assay to determine EhRNaseIII function. *In vitro* assays are a classic method to demonstrate substrate cleavage and have been used to prove the functionality of numerous Dicer enzymes in varied systems including *T*. *brucei*, *Giardia intestinalis*, *Drosophila melanogaster*, *Schizosaccharomyces pombe*, and the budding yeast *Saccharomyces castellii* [17–21, 37, 38].

**Fig 2:**
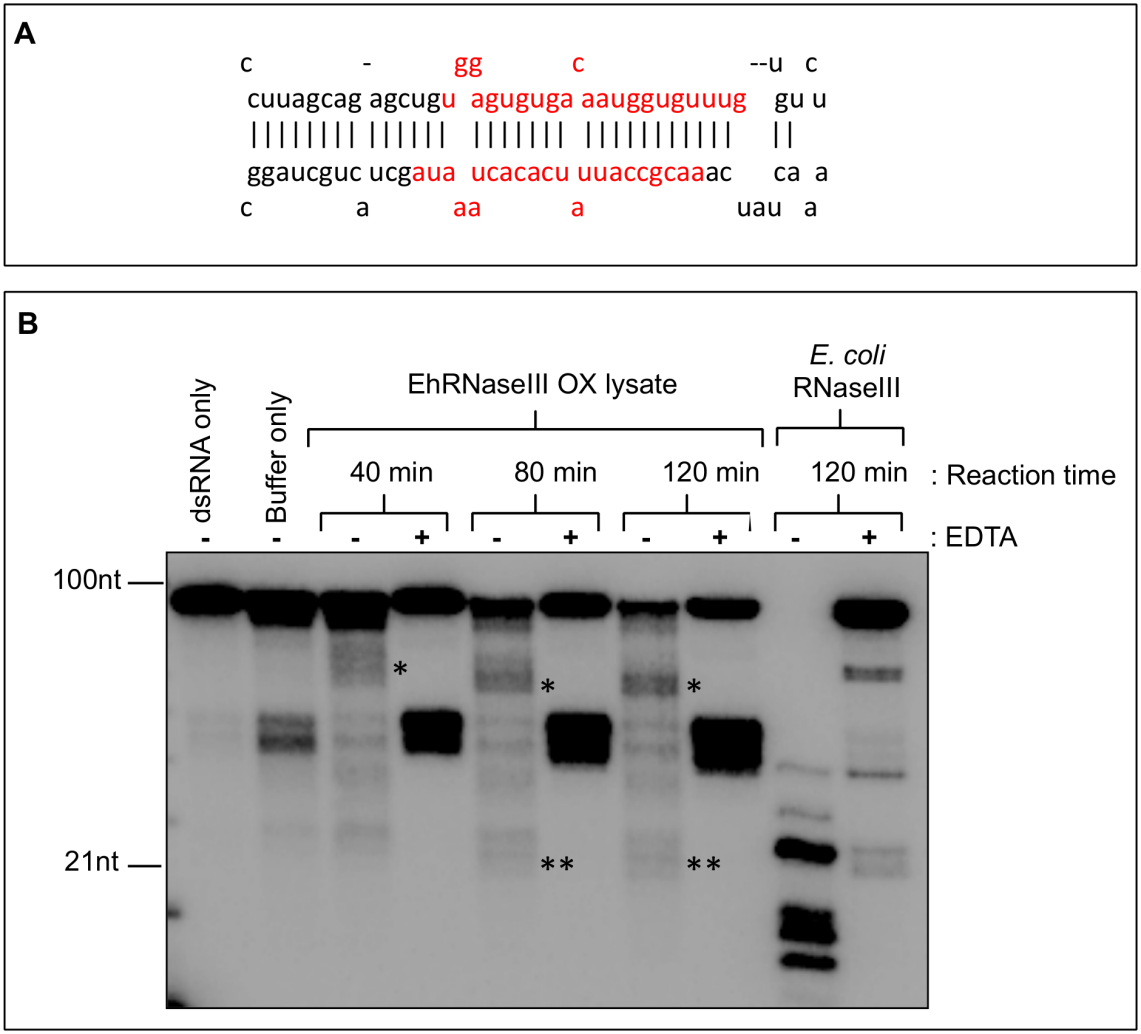
Limited RNaseIII activity in *E*. *histolytica* trophozoite lysate. **(A)** Stem-loop structure of pre-miR122 dsRNA substrate used for RNaseIII activity assay. Mature miR-122/miR-122* duplex shown in red. Structure obtained from http://www.mirbase.org/cgi-bin/mirna_entry.pl?acc=MI0000442. **(B)** *In vitro* cleavage of [α-^32^P]ATP-labeled pre-miR122 by 10μl of whole cell lysate from *E*. *histolytica* trophozoites expressing Myc-EhRNaseIII. Reactions contained 3mM MgCl_2_, 20mM potassium glutamate, ± 20mM EDTA and were incubated for 40, 80, or 120 minutes at 37°C. EDTA chelates the magnesium and inhibits RNaseIII activity. The dsRNA only sample contained no protein. The buffer only sample contained 10μl of lysate buffer (no protein). dsRNA and buffer only samples were incubated for 120 minutes at 37°C. Positive control reactions contained 1U of *E*. *coli* RNaseIII (Ambion). Asterisks indicate specific cleavage products– 50-60nt (*) and 20-21nt (**). The 40-50nt bands are non-specific products of the pre-miR122 cleavage.

Abed *et al*. previously reported RNaseIII activity *in vitro* in *E*. *histolytica* lysate with degradation of the dsRNA substrate within one hour and the appearance of a diffuse approximately 100bp product after a 16 hour incubation [27). We were able to recapitulate these findings in a similar *in vitro* cleavage assay using a pre-microRNA dsRNA substrate (pre-miR122), shorter incubation times, and whole cell lysate from trophozoites constitutively overexpressing EhRNaseIII (Fig 2). Limited RNA processing was observed resulting in specific products of approximately 50-60nt and faint products around 20-21nt (Fig 2B). Addition of EDTA chelates the divalent cation, which is required for RNA hydrolysis and consequently inhibits RNaseIII activity [16]. Interestingly, in the presence of EDTA, we were not able to detect processed products, suggesting that these products are specific results of dsRNA processing. Abundant 40-50nt non-specific products were detected despite the addition of EDTA likely due to the efficient cleavage of the imperfect stem-loop structure of pre-miR122. Recombinant *Escherichia coli* RNaseIII efficiently cleaved the pre-miR122 substrate into specific products (Fig 2B). The use of cold single-stranded RNA and recombinant EhRNaseIII in cleavage reactions did not reduce substrate degradation (data not shown). Cleavage assays using whole cell lysate with endogenous levels of Dicer have been successful in other systems [18–20]. However, it is likely that *E*. *histolytica* contains too many nucleases to allow for ample accumulation of small RNA cleavage products *in vitro* using whole cell extract or alternatively the EhRNaseIII enzyme may be of low efficiency leading to low level specific dsRNA cleavage.

We employed a less readily hydrolyzed substrate—a 125bp perfect duplex dsRNA (EhdsRNA) from EHI_165450, a gene with abundant endogenous 27nt antisense RNAs in *E*. *histolytica* trophozoites [15]. Extensive degradation was still observed with whole cell lysate from untransfected trophozoites (S2 Fig) and using smaller volumes of lysate from parasites overexpressing EhRNaseIII did not improve degradation (S2 Fig). Seeking to further minimize degradation and enrich for EhRNaseIII activity, we immunoprecipitated EhRNaseIII from EhRNaseIII-overexpressing parasites under a variety of wash conditions, yet no cleavage was detected (data not shown). The budding yeast Dicer *S*. *castellii* Dcr1 resembles EhRNaseIII in structure and is sufficient to generate sRNAs *in vitro* [20]; therefore, we also assessed whether recombinant EhRNaseIII alone was capable of cleaving dsRNA *in vitro*. No RNA processing was observed under a range of magnesium chloride or salt concentrations (data not shown). Supplementing both recombinant protein and immunoprecipitation reactions with an additional divalent cation, calcium chloride, and extending reaction times to 24 hours did not yield specific cleavage products (data not shown). Collectively, these data suggest that either EhRNaseIII does not cleave dsRNA in the presence of magnesium or calcium or that the optimal conditions for EhRNaseIII immunoprecipitation and/or cleavage of dsRNA *in vitro* have not been identified. It is also possible that a cofactor, such as a dsRNA-binding protein, is needed for the dsRNA processing activity of EhRNaseIII.

### EhRNaseIII and EhAgo2-2 do not reconstitute the RNAi pathway in *S*. *cerevisiae*

**Fig 3:**
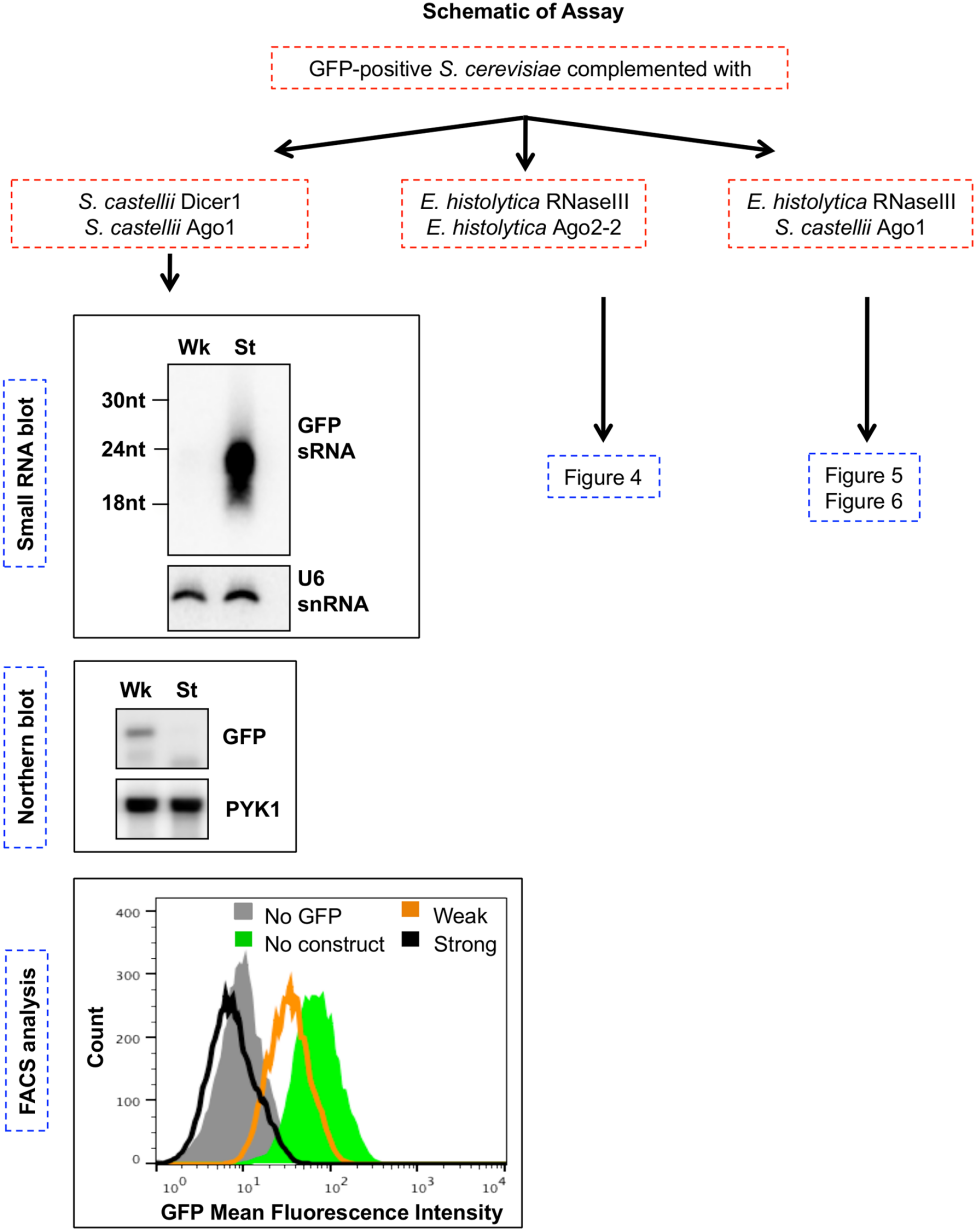
Schematic of RNAi reconstitution assays in *S*. *cerevisiae*. Flow chart of experiments in which GFP-positive *S*. *cerevisiae* was transformed with *S*. *castellii* or *E*. *histolytica* genes. Expression of the weak (Wk) silencing construct (long GFP dsRNA) or the strong (St) silencing construct (GFP hairpin) was induced in each strain. For each strain listed, high resolution small RNA Northern blots, total RNA Northern blots, and flow cytometry (FACS) analysis were performed. Northern blots and FACS histogram data shown refer to *S*. *cerevisiae* strains expressing *S*. *castellii* Dicer1 and *S*. *castellii* Ago1 and either the weak (Wk) or the strong (St) GFP silencing construct. These data recapitulate findings from [20].

**Fig 4:**
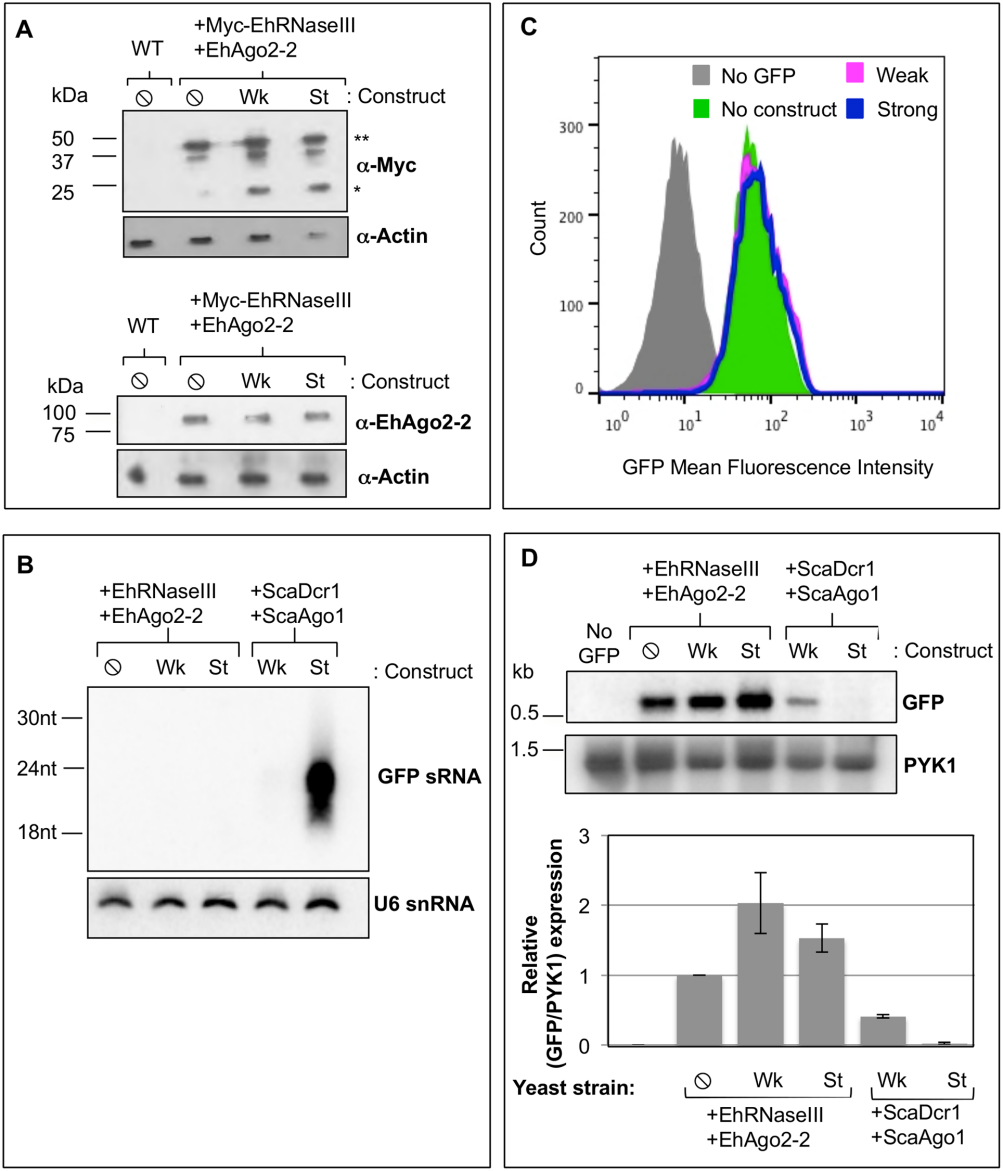
EhRNaseIII and EhAgo2-2 do not mediate silencing of GFP. GFP-positive *S*. *cerevisiae* untransformed (WT) and transformed with Myc-EhRNaseIII and EhAgo2-2 and expressing no GFP silencing construct (⦸), weak GFP long dsRNA silencing construct (Wk), or the strong GFP hairpin silencing construct (St). **(A)** Western blot from lysate of indicated strains probed with α-Myc, α-EhAgo2-2, or α-actin shows that EhRNaseIII and EhAgo2-2 proteins are expressed in all transformed cell lines. *: EhRNaseIII monomer; **: EhRNaseIII dimer. **(B)** High resolution Northern blot probed for GFP sRNAs in the strains listed in (A) indicates that EhRNaseIII does not generate abundant GFP sRNAs. *S*. *cerevisiae* expressing *S*. *castellii* Dicer1 and Ago1 serve as positive controls. Blot was stripped and re-probed for U6 small nucleolar RNAs as a loading control. **(C)** FACS histogram of GFP fluorescence in the indicated *S*. *cerevisiae* strains shows that EhRNaseIII and EhAgo2-2 are unable to silence GFP using either the weak or strong GFP silencing RNA substrates. **(D)** Upper: Northern blot probed for GFP mRNA in the indicated *S*. *cerevisiae* strains shows no decrease in GFP transcript in strains expressing EhRNaseIII and EhAgo2-2 and either the weak or strong GFP silencing construct. Strain DPB271 (no GFP) was used as negative control. Blot was stripped and re-probed for PYK1 as a loading control. Lower: Quantitation of GFP band intensities normalized by PYK1 expression. The corresponding values are relative to GFP expression in the absence of silencing constructs. Two experiments represented.

Given the difficulties with assessing EhRNaseIII activity *in vitro*, we turned to a heterologous system to probe the function of EhRNaseIII (Fig 3). Drinnenberg and colleagues showed that RNAi pathway components from an RNAi (+) budding yeast system are sufficient to reconstitute RNAi gene silencing in an RNAi (-) budding yeast [20]. Thus, *S*. *castellii* Dicer (ScaDcr1) and *S*. *castellii* Argonaute (ScaAgo1) are sufficient to reconstitute RNAi gene silencing in *S*. *cerevisiae*, an RNAi-deficient system [20]. We were able to recapitulate these data in our laboratory (Fig 3). This reconstitution system is based on three GFP-positive *S*. *cerevisiae* strains stably transformed with either an inducible weak silencing construct that produces long GFP dsRNA, an inducible strong silencing construct that produces a GFP hairpin RNA, or no GFP silencing construct [20]. A similar study using human RNAi proteins—Dicer, Ago2, and HIV-1 transactivating response RNA-binding protein (TRBP)—demonstrated that the reconstitution approach is broadly applicable across eukaryotic systems [39]. To determine whether *E*. *histolytica* Ago2-2 (EhAgo2-2) and EhRNaseIII are able to reconstitute the RNAi pathway in *S*. *cerevisiae*, we expressed EhAgo2-2 and EhRNaseIII in *S*. *cerevisiae* and assayed for generation of GFP sRNAs as well as for silencing of GFP transcript and protein. To ensure that the amebic proteins would be expressed, we codon-optimized EhRNaseIII and EhAgo2-2 for expression in *S*. *cerevisiae* and integrated these genes into the chromosomes of the three reporter strains. We added an N-terminal 3xMyc tag to the codon-optimized EhRNaseIII gene to verify expression of this protein in the transformed strains. As shown by Western blot analysis, both Myc-EhRNaseIII and EhAgo2-2 were expressed in all three *S*. *cerevisiae* reporter strains (Fig 4A). Two species of EhRNaseIII were detected—a 50 kDa and 25 kDa band (Fig 4A). The 25 kDa size is consistent with an EhRNaseIII monomer, suggesting that the 50 kDa band is a dimer. Given the denaturing lysate preparations, the presence of the larger band may suggest that EhRNaseIII forms a tight dimer, which is consistent with RNaseIII mechanism of action [16].

To ascertain whether EhRNaseIII could generate sRNAs to GFP, we grew the Myc-EhRNaseIII/EhAgo2-2 expressing reporter strains in galactose to induce production of the GFP silencing substrates and assayed for sRNA generation by high resolution Northern blot analysis. Strains expressing ScaDcr1/ScaAgo1 were included as positive controls. As expected, abundant GFP sRNAs were detected in the control ScaDcr1/ScaAgo1 strain expressing the strong silencing substrate. However, no GFP sRNAs were detected in the EhRNaseIII reporter strains expressing either the weak or the strong silencing dsRNA substrate (Fig 4B). Notably, the ScaDcr1/ScaAgo1 strain expressing the weak silencing construct also does not show abundant GFP sRNA generation by Northern blot analysis but GFP is partially silenced in this strain indicating that sRNAs were produced ([20], Fig 4D). Therefore, it is possible that EhRNaseIII produces GFP sRNAs but which are below the limit of detection of by Northern blot analysis.

We next examined GFP protein and mRNA levels to assess whether EhRNaseIII generated a smaller pool of GFP sRNAs than can be assayed by Northern blot analysis, but which are adequate and functional to reduce GFP expression. Flow cytometry was used to measure GFP fluorescence in strains expressing EhRNaseIII/EhAgo2-2 and in strains expressing ScaDcr1/ScaAgo1. A GFP-negative *S*. *cerevisiae* strain that contained no RNAi genes was used as a negative control and the GFP-positive reporter strain expressing EhRNaseIII/EhAgo2-2 but lacking a silencing construct served as a positive control for GFP expression. The same culture samples were used for Northern blot and flow cytometry analyses. Drinnenberg and colleagues showed that ScaDcr1/ScaAgo1 strains exhibit a strong shift in GFP fluorescence with the strong silencing substrate, in accordance with the abundant GFP sRNAs produced by ScaDcr1 [20]. They also demonstrated an intermediate shift in fluorescence with the weak silencing substrate indicating that GFP sRNAs are produced from long dsRNA even though they are not detected by Northern blot analysis [20]. However, in our assays with the strains expressing EhRNaseIII/EhAgo2-2 and either the weak or the strong silencing substrate there was no shift in GFP fluorescence (Fig 4C), although we were able to recapitulate the positive results with ScaDcr1/ScaAgo1 (Fig 3). These results were corroborated by the Northern blot data for GFP mRNA where there was no decrease in transcript levels among the strains expressing EhRNaseIII/EhAgo2-2 (Fig 4D). In fact, there was a slight increase in GFP transcript in strains expressing a silencing construct (Fig 4D), potentially a stress response to expressing but not processing the dsRNA substrates. Taken together, these data indicate that EhRNaseIII and EhAgo2-2 are not able to reconstitute the RNAi pathway in *S*. *cerevisiae*.

These data may indicate that EhRNaseIII does not generate sRNAs. Alternatively, the lack of functional GFP silencing could be due to the inability of EhAgo2-2 to bind and/or recognize sRNAs that are characteristic of RNaseIII cleavage. Dicer-generated sRNAs have characteristic 5′-monophosphate termini [14, 16, 40]. A conserved binding pocket in Ago proteins mediates specificity for 5′ features of sRNAs and thus Argonaute proteins bind sRNAs with either 5′-monophosphate or 5′-polyphosphate termini [41]. Given that EhAgo2-2 endogenously associates with 5′-polyphosphate sRNAs in *E*. *histolytica* [13], this protein may not be able to bind and/or mediate silencing using 5′-monophosphate sRNAs generated in *S*. *cerevisiae*. Thus, the lack of GFP silencing in *S*. *cerevisiae* with EhRNaseIII/EhAgo2-2 may be due to the inability of EhRNaseIII to cleave dsRNA or instead may be due to the inability of EhAgo2-2 to use 5′-monophosphate sRNAs for gene silencing (through either an inability to bind or to mediate target RNA silencing using 5′-monophosphate sRNAs).

### EhRNaseIII and ScaAgo1 mediate partial silencing of GFP

**Fig 5:**
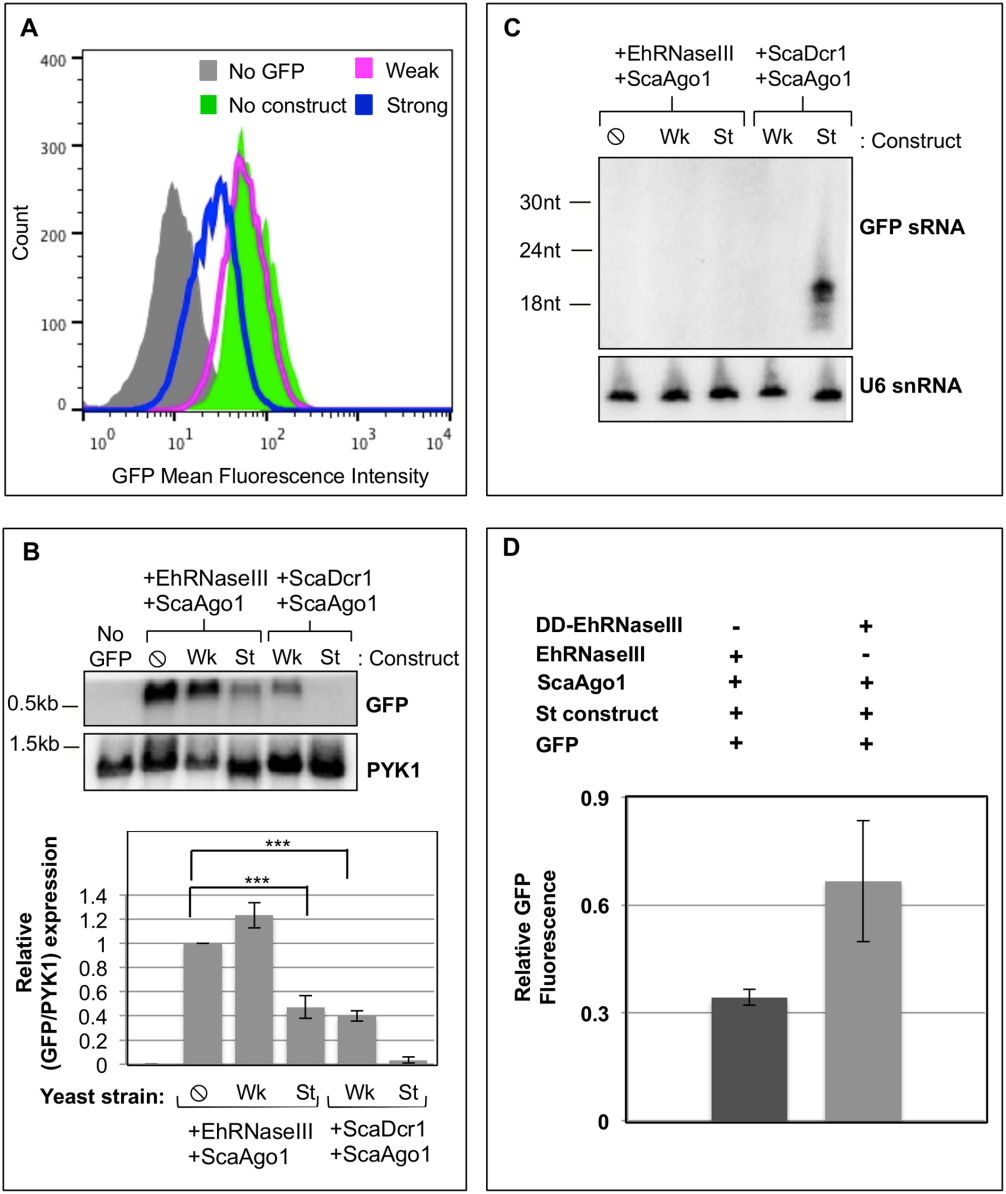
EhRNaseIII and ScaAgo1 mediate partial silencing of GFP. GFP-positive *S*. *cerevisiae* transformed with Myc-EhRNaseIII and *S*. *castellii* Ago1 expressing no silencing construct (⦸), the weak GFP long dsRNA silencing construct (Wk), or the strong GFP hairpin silencing construct (St). **(A)** FACS histogram of GFP fluorescence in the indicated strain shows decrease in GFP fluorescence in the strain expressing EhRNaseIII and ScaAgo1 using the strong GFP silencing hairpin. **(B)** Upper: Northern blot of total RNA from the indicated strains shows a decrease in GFP transcript in the EhRNaseIII/ScaAgo1 strain with the strong silencing construct. Lower: Quantitation of GFP band intensities normalized by PYK1 expression. The corresponding values are relative to GFP expression in the absence of silencing constructs. Three experiments represented. *** indicates statistical significance with p<0.05 by t-test. **(C)** High resolution Northern blot using 15μg of total RNA from indicated strains probed for GFP sRNAs. Blot was stripped and re-probed for U6 small nucleolar RNAs. **(D)** Mean GFP intensity is plotted as a fraction of GFP expressing yeast strains lacking silencing construct but containing EhRNaseIII or domain disruption EhRNaseIII (DD-EhRNaseIII). P<0.05. Three independent experiments are represented. Domain disruption EhRNaseIII contains amino acids 1–133 of EhRNaseIII fused to the C-terminal domain (amino acids 265–610) of *S*. *castellii* Dcr1.

One possibility is that EhRNaseIII is capable of generating GFP sRNAs that are below the limit of detection by Northern blot analysis and cannot load into EhAgo2-2. In order to assess this possibility, we utilized ScaAgo1, which is capable of binding 5′-monophosphate sRNAs and using them to mediate gene silencing [20]. We generated *S*. *cerevisiae* GFP reporter strains expressing EhRNaseIII and ScaAgo1. Using the weak and strong silencing constructs, we determined the level of GFP expression in these strains. In contrast to all previous results, reconstitution of *S*. *cerevisiae* with EhRNaseIII and ScaAgo1 revealed a reduction in GFP fluorescence in cells induced with the strong silencing construct, whereas no GFP shift was noted with the weak silencing construct (Fig 5A). The GFP reduction with EhRNaseIII/ScaAgo1 and the strong silencing construct resembled the intermediate silencing phenotype observed with ScaDcr1/ScaAgo1 and the weak silencing construct. Northern blot analysis confirmed the flow cytometry data of intermediate GFP reduction. GFP transcript abundance was reduced to similar levels in both the EhRNaseIII/ScaAgo1 strong construct strain and the ScaDcr1/ScaAgo1 weak construct strain (Fig 5B). However, despite GFP reduction of both transcript and protein levels, no GFP sRNAs were detected by high resolution Northern blot analysis with 15μg of total RNA in either the EhRNaseIII/ScaAgo1 strong silencing construct strain or the ScaDcr1/ScaAgo1 weak silencing construct strain (Fig 5C). These data indicate that in an *S*. *cerevisiae* reconstitution system, EhRNaseIII produces GFP sRNAs that are capable of mediating silencing but in quantities that are not abundant enough to be detected by Northern blot analysis, similar to the outcome noted for ScaDcr1/ScaAgo1 with the weak silencing construct.

Many Dicer enzymes contain domains that bind dsRNA [17, 20, 21, 42, 43]. Deletion analysis of the *Kluyveromcyes polysporus* budding yeast Dicer showed that at least one dsRBD is required for efficient dsRNA cleavage and for accurate sRNA length determination *in vitro* [21]. However, studies with *S*. *castellii* Dicer showed that two dsRBDs are required for sRNA accumulation *in vivo* [21]. To determine whether EhRNaseIII-directed cleavage and silencing in *S*. *cerevisiae* could be altered by the addition of dsRBDs, we generated a chimera between EhRNaseIII and ScaDcr1. The C-terminal region of EhRNaseIII (residues 134–256) was replaced with the C-terminal region of ScaDcr1 (residues 265–610) that contains the two dsRBDs. We generated *S*. *cerevisiae* GFP reporter strains expressing the EhRNaseIII-dsRBD fusion protein and ScaAgo1 and assayed for GFP silencing by flow cytometry using the strong silencing substrate. We confirmed that the hybrid RNaseIII protein was stably expressed but rather than improve EhRNaseIII-mediated cleavage, this fusion protein exhibited reduced cleavage ability demonstrated by an increase in GFP fluorescence compared to wild type EhRNaseIII (Fig 5D). Thus, we postulate that the C-terminal truncation and/or the addition of the *S*. *castellii* dsRBDs altered the conformation of the *E*. *histolytica* RNaseIII domain, effectively resulting in a domain disruption and a less efficient enzyme. These data support our findings that EhRNaseIII function contributes to gene silencing in the *S*. *cerevisiae* system. These data also indicate that there may be heretofore unrecognized important features in the C-terminal region of EhRNaseIII, which contributes to its dsRNA processing activity.

### EhRNaseIII is capable of processing dsRNA to smaller fragments that mediate gene silencing in association with ScaAgo1

**Fig 6:**
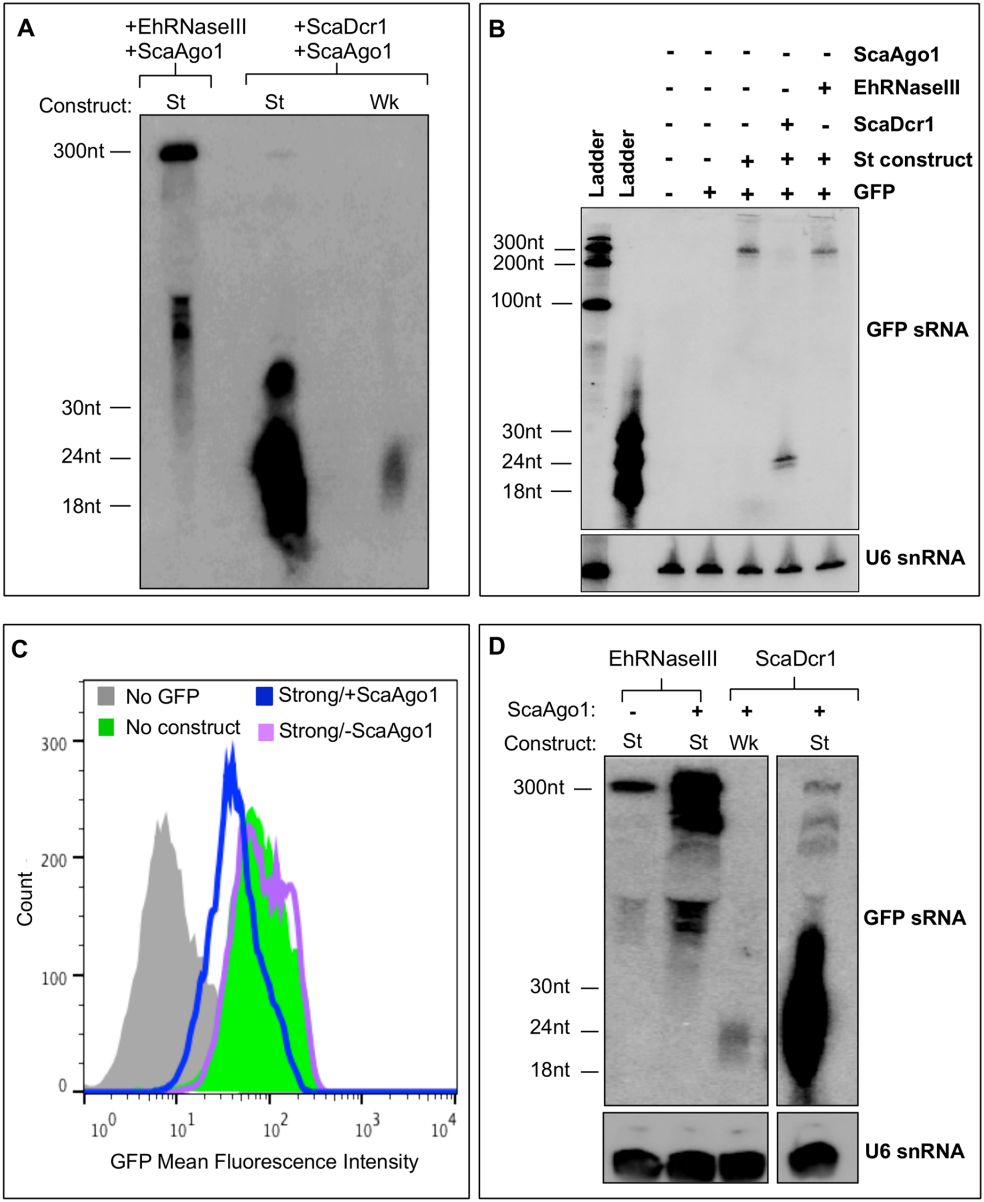
Partial silencing of GFP requires both EhRNaseIII and ScaAgo1. **(A)** *S*. *cerevisiae* expressing Myc-EhRNaseIII and *S*. *castellii* Ago1 and the strong GFP silencing construct (St). *S*. *cerevisiae* expressing ScaDcr1 and ScaAgo1 and either the weak (Wk) or Strong (St) GFP silencing construct. High resolution Northern blot probing 50μg of sRNA-enriched material from indicated strains for GFP sRNAs. 300nt and ~50-60nt RNA species detected in EhRNaseIII/ScaAgo1/St cell line. **(B)** Northern blot of 15μg of total RNA from the indicated strains shows 300nt band corresponds to unprocessed strong silencing GFP hairpin that is completely processed into sRNAs in presence of ScaDcr1. RNA resolved by 8M urea, 12% polyacrylamide gel electrophoresis **(C)** *S*. *cerevisiae* expressing the strong GFP silencing construct and EhRNaseIII with or without ScaAgo1. FACS histogram of GFP fluorescence in the indicated strains show a decrease in GFP fluorescence in strain expressing EhRNaseIII using the strong GFP silencing hairpin but only in the strain expressing ScaAgo1. **(D)** High resolution Northern of 100μg of sRNA-enriched material from indicated strains probed for GFP sRNAs. Blot was stripped and re-probed for U6 small nucleolar RNAs. Blot shows ~50-60nt processed RNA species in presence of EhRNaseIII, the abundance of which increases with the addition of ScaAgo1. In the presence of ScaDcr1/ScaAgo1, the weak and strong silencing constructs are both efficiently processed to ~20-30nt small RNAs.

Given that EhRNaseIII/ScaAgo1 resulted in reduction of GFP, we sought to better visualize EhRNaseIII-generated GFP sRNAs by probing 50μg of sRNA-enriched material in a high resolution Northern blot analysis. With the use of sRNA-enriched material, GFP sRNAs produced from the weak RNA substrate by ScaDcr1 were visible for the first time (Fig 6A). In the EhRNaseIII/ScaAgo1 strain, we could also detect two RNA species: the first at ~300nt and the second as a diffuse population at ~50-60nt (Fig 6A). To determine the origin of the 300nt and 50-60nt RNA species, we probed 15μg of total RNA from different strains for GFP sRNAs (Fig 6B). The strains we tested lacked ScaAgo1 but contained combinations of GFP, the strong silencing construct, and ScaDcr1 or EhRNaseIII. The 300nt RNA species corresponds to the denatured strong silencing hairpin and in the presence of ScaDcr1 was processed completely into GFP sRNAs (Fig 6B). In the presence of EhRNaseIII, the 300nt RNA was processed into the smaller 50-60nt RNA fragments with diffuse processing to smaller fractions, but without the production of discrete sRNAs (Fig 6A). The 50-60nt population is specific to the presence of EhRNaseIII and thus represent EhRNaseIII-specific processing.

The GFP sRNAs generated by EhRNaseIII suggest that EhRNaseIII may use a different mechanism to determine sRNA length and/or an amebic factor is needed for complete and efficient dsRNA processing. The expected length of sRNAs produced by EhRNaseIII and how EhRNaseIII determines the length of its sRNAs are not known. Budding yeast Dicers generate 23nt sRNAs; this is the distance between the active sites of adjacent Dicer dimers that assemble on the dsRNA substrate [20, 21]. Despite the larger size of the sRNAs produced by EhRNaseIII in the *S*. *cerevisiae* system, these sRNAs are capable of partially silencing GFP and appear to be as efficient as the GFP sRNAs produced by ScaDcr1 using the weak RNA substrate (Fig 5B). Further investigation is required to identify proteins that may interact with EhRNaseIII to mediate highly efficient processing. How ScaAgo1 utilizes the larger EhRNaseIII-generated sRNAs to silence GFP is also unclear. The crystal structure of another budding yeast Ago, *Kluyveromyces polysporus* Ago, reveals an extended, unobstructed nucleic acid binding channel [44]. Perhaps this conformation allows for binding of the longer EhRNaseIII sRNAs. Another possibility is that EhRNaseIII sRNAs are trimmed by the endogenous *S*. *cerevisiae* RNaseIII, Rnt1, which processes pre-ribosomal RNAs, small nuclear RNAs, and small nucleolar RNAs [45], to a length which ScaAgo1 can bind.

To test specificity of the results of the EhRNaseIII/ScaAgo1 strain we chose to analyze the parental strain that contains the GFP reporter and the strong silencing construct and expresses EhRNaseIII but not ScaAgo1. First, we used flow cytometry to examine GFP expression. As expected, EhRNaseIII without ScaAgo1 showed no shift in GFP fluorescence (Fig 6C). Drinnenberg *et al*. have previously shown that ScaAgo1 does not mediate GFP silencing in the absence of ScaDcr1 [20] and thus the partial silencing with EhRNaseIII/ScAgo1 should not be attributed to ScAgo1. Thus, the GFP reduction seen with EhRNaseIII/ScaAgo1 should be due to EhRNaseIII-generated sRNAs but is dependent on the presence of ScaAgo1 to mediate silencing (Fig 6C). In order to visualize low abundant sRNA populations, we probed 100μg of sRNA-enriched material for GFP sRNAs. We could not detect discrete sRNAs in the 20-30nt range in the presence of EhRNaseIII/ScaAgo1 but again observed the larger RNA processing intermediates (Fig 6D). Of note, the pattern of processed RNA for the ~100-300nt fragments is similar to that of ScaDcr1/ScaAgo1. The EhRNaseIII/strong silencing construct strain, which lacks ScaAgo1, showed a lower abundance of processed RNA suggesting that ScaAgo1 may facilitate stabilizing the sRNA products (Fig 6D). In other systems, Ago is important for sRNA stability and escorting sRNAs to their final destination to base pair with their cognate mRNA [44, 46]. Collectively, these data indicate that EhRNaseIII is capable of processing dsRNA into smaller fragments that can partially silence a gene target—a function reminiscent of Dicer enzymes. Thus, even though EhRNaseIII lacks the canonical Dicer structure, it possesses some Dicer-like activity, which in conjunction with an Argonaute protein is able to mediate RNA processing and gene silencing.

## Discussion

Small RNAs mediate gene silencing in the RNAi pathway with Dicer, an RNaseIII enzyme, being central to the majority of small RNA generation pathways. Although many Dicer enzymes have complex domain architecture, structural minimalism of this protein has increasingly been reported. In this study we have identified a unique and very minimal Dicer candidate in the protist *Entamoeba histolytica*. We demonstrated that although amebic EhRNaseIII contains only a single RNaseIII domain and lacks all other recognizable domains, it is capable of processing dsRNA into shorter RNA fragments that, in combination with an Argonaute protein, can mediate gene silencing in a heterologous system. Overall, these data indicate that EhRNaseIII has the potential to operate as a Dicer-like enzyme in *Entamoeba* and may expand the repertoire of non-canonical proteins that contribute to the RNAi pathway in non-model systems.

Studying EhRNaseIII in an RNAi-negative background revealed its dsRNA processing ability that was otherwise confounded in *in vitro* studies. The incomplete silencing of GFP in *S*. *cerevisiae* suggests that processing of dsRNA substrates by EhRNaseIII could be enhanced by amebic cofactors. Notably absent from EhRNaseIII are domains commonly found in Dicer enzymes that bind dsRNA such as PAZ or dsRBDs. Dicer enzymes in other systems often associate with dsRNA binding proteins such as TRBP, PACT, Loquacious, RDE-4, and R2D2, although not all of them are required for small RNA generation [33, 43, 47–49]. Perhaps the addition of a dsRNA binding protein would help EhRNaseIII achieve maximal RNaseIII activity either through enhanced binding of the dsRNA substrate, proper positioning, and/or coordinated cleavage of the substrate. It is also possible that EhRNaseIII activity is normally low and that additional amebic factors are required to amplify the low abundant small RNAs generated by EhRNaseIII. Importantly, in *E*. *histolytica* secondary small RNAs with 5'-polyphosphate termini are abundant and in systems with amplified silencing such as nematodes, these secondary small RNAs likely arise from RNA-dependent RNA polymerase (RdRP) activity [9, 10, 13]. *E*. *histolytica* has two RdRP genes and thus an inefficient Dicer-like enzyme may be counterbalanced by active RdRP-mediated generation of secondary small RNAs leading to efficient gene silencing [26].

The mechanism of how EhRNaseIII determines the size of its products is unclear as it contains no defined RNA binding domain. In canonical Dicer enzymes, small RNA length is determined by the distance from the PAZ domain to the catalytic center of the RNaseIII domains [17, 50]. There is evidence that some Dicer enzymes, such as human and *Drosophila* Dicer, also measure from the 5' end of the dsRNA rather than from the 3'-overhang that binds the PAZ domain [51]. Thus, canonical Dicers measure from the ends of their dsRNA substrates. Budding yeast Dicers, however, utilize an “inside-out” mechanism where cooperative binding of the two Dicer dimers occurs randomly at an internal site on the dsRNA. The distance between the active sites of adjacent dimers determines the size of the small RNA and processing continues outward towards the ends of the dsRNA [21]. In the extremely pared down structure of EhRNaseIII, it is plausible that additional amebic proteins are required to coordinate dsRNA binding and to generate small RNAs of a specific length. The range of processed RNA visible in the EhRNaseIII/ScaAgo1 strain may be due to the lack of other amebic RNAi protein machinery in *S*. *cerevisiae*. Identification of proteins that associate with EhRNaseIII may help elucidate the molecular ruler utilized by the amebic “Dicer-like” complex.

Building upon the existing knowledge of the amebic RNAi pathway as well as the RNAi pathway in other systems, we propose a new working model of the RNAi pathway in *E*. *histolytica*. EhRNaseIII in conjunction with other amebic cofactors forms a Dicer-like complex. This complex processes structured dsRNA precursors into shorter fragments. Drawing on similarities with the *C*. *elegans* RNAi pathways, we would expect that these Dicer-derived primary small RNAs are rare, which would explain the lack of an abundant endogenous 5'-monophosphate small RNA population in *E*. *histolytica* trophozoites [13]. According to the current model of how Argonaute proteins recognize and bind their small RNAs, we would expect that EhAgo2-2 is not involved in binding amebic EhRNaseIII-derived small RNAs since it associates with 5'-polyphosphate small RNAs. This preference for 5'-polyphosphate small RNAs would also explain why EhRNaseIII in concert with EhAgo2-2 was unable to reconstitute the RNAi pathway in *S*. *cerevisiae*. A number of outstanding questions are raised by our data including: What other proteins comprise the Dicer-like complex in *Entamoeba*? What is the size of Dicer-derived small RNAs in ameba? And how does the complex determine the length of its products? Future investigations will focus on answering some of these questions with the priority being identifying other components of the Dicer-like complex. In summary, this work demonstrates that *E*. *histolytica* RNaseIII contributes to small RNA biogenesis in *Entamoeba* and likely gene silencing via the RNAi pathway.

## Materials and Methods

### Parasite culture, generation of transgenic parasite strains, and plasmid construction

*E*. *histolytica* HM-1:IMSS parasites were grown axenically under standard conditions [52, 53]. Mid-log trophozoites were transfected with 20μg of purified plasmid using 30μl of Superfect Reagent (Qiagen) according to standard protocols [54]. Stable transfectants were selected at 3μg/ml G418 and maintained at 12μg/ml G418. EhRNaseIII (EHI_068740) was amplified from HM-1:IMSS genomic DNA using forward and reverse primers (F- CCCGGGAGCTCAACTACATTA, R- CTCGAGTTATTGTGATGGATGAAC) containing SmaI and XhoI restriction sites, respectively. The PCR product was subcloned into pCR2.1-TOPO (Life Technologies), digested with SmaI and XhoI, and cloned into pKT-3M (kind gift of Dr. Tomo Nozaki, National Institute of Infectious Disease, Japan), which contains an N-terminal 3xMyc tag.

### Bioinformatics analysis

Pfam profile HMMs for the RNaseIII domain (PF00636) and the PAZ domain (PF02170) were retrieved from Pfam_ls HMMs (version 23). The HMMER program (version 2.3.2) was used to build custom profile HMMs for RNaseIII and PAZ domains using clustalW multiple sequence alignments from sequences in S2 and S3 Tables, respectively. EhRNaseIII (EHI_068740) and the PAZ domain from EhAgo2-2 (EHI_125650; amino acids 201–337) were manually added to the appropriate sequence files before generation of the multiple sequence alignment. The *E*. *histolytica* genome (JCVI data release 5.0) was translated in six-frames. All HMMs were searched against both the translated and annotated genomes using HMMER and hits with e-values ≤ 0.5 were retained.

### dsRNA and sample preparation and in vitro cleavage assays

Pre-miR122: The pGL3-pre-miR-122 plasmid (kind gift of Dr. Gabriele Fuchs, Stanford University) was linearized with SfoI and *in vitro* transcribed using the MEGAshortscript T7 kit (Life Technologies) with a 6000:1 molar ratio of ATP:[α-^32^P]ATP (800 Ci/mmol) according to the manufacturer’s instructions. An 81nt product was excised from an 8% polyacrylamide, 8M urea gel and eluted overnight at room temperature in 0.5M ammonium acetate, 1mM EDTA, and 0.1% SDS adjusted to pH 5.0 with acetic acid. The RNA was extracted with an equal volume of acid-phenol:chloroform (Ambion), then extracted with an equal volume of chloroform:isoamyl alcohol (24:1), and ethanol precipitated without extra salt. Pre-miR-122 was heated at 65°C for 5 minutes and gradually cooled to room temperature. The structure of pre-miR122 can be found at http://www.mirbase.org/cgi-bin/mirna_entry.pl?acc=MI0000442.

EhdsRNA (duplex substrate): The first 125bp of EHI_165450 were amplified from HM-1:IMSS genomic DNA using the forward and reverse primers (F- TTATACGGTACCCCTAGGATGAGTGACATCAACAACAAC, R- AAATGTAAGATGCGGCCGCGGCGCCTCCTCTTCGGCTAC) containing KpnI and NotI restriction sites, respectively and cloned into pBSII KS+ (Stratagene). One strand was *in vitro* transcribed using the MEGAshortscript T7 kit (Life Technologies) with a 6000:1 molar ratio of ATP:[α-^32^P]ATP (800 Ci/mmol) and the other strand was transcribed using MEGAscript T3 kit (Life Technologies) according to the manufacturer’s instructions. Both strands were transcribed from the same plasmid linearized with SfoI or AvrII (for T7 and T3, respectively). Strands were gel purified and annealed with 20-fold excess of the cold strand by heating at 95°C for 2 minutes and slow cooling to room temperature.

Whole cell extracts were prepared from 7x10^6^ mid-log *E*. *histolytica* trophozoites as described in [55], flash frozen and stored in aliquots at -80°C. Dicing assays were conducted in 20μl volumes where 10μl of whole cell extract was incubated with 10,000 cpm of radiolabeled dsRNA (~2 ng/μl final concentration) in reaction buffer (150mM sucrose, 20mM potassium L-glutamate (Sigma), 20mM HEPES-KOH, pH 7.9, 3mM MgCl_2_, 10 μg/ml leupeptin, 1 Complete Mini EDTA-free protease inhibitor cocktail tablet (Roche Diagnostics) for every 3ml of buffer) [19] supplemented with 30U Protector RNase Inhibitor (Roche Diagnostics) and 1U/μl SUPERNase·IN RNase Inhibitor (Ambion) with or without 20mM EDTA for 2 hours at 37°C unless otherwise indicated. 1μl of recombinant *E*. *coli* RNaseIII (Ambion) was used as a positive technical control. Reactions were quenched with 30mM EDTA and RNA was extracted with acid-phenol:chloroform, desalted with Micro Bio-Spin 6 columns (BioRad), and ethanol precipitated. RNA was resolved on a denaturing 10% acrylamide sequencing gel, dried and analyzed by Personal Molecular Imager (BioRad) and Quantity One (BioRad) where contrast was adjusted across the entire image.

### SDS-PAGE and Western blot analysis

For *S*. *cerevisiae* samples, 5 ODU (at OD_600_) of cells were solubilized in SUME buffer (1% SDS, 8M Urea, 10mM MOPS, pH 6.8, 10mM EDTA, 0.01% bromophenol blue) supplemented with protease inhibitors (10mM PMSF, 100μg/ml TPCK, 100μg/ml leupeptin), beat with acid-washed glass beads (Sigma) and heated at 65°C for 10 min. Proteins resolved by 12% PAGE, transferred to PVDF membrane (BioRad). Anti-Myc antibody (Santa Cruz Biotechnology, Inc.) was used to detect EhRNaseIII in JMP019-027 strains and anti-EhAgo2-2 (1:1000) from [14] was used to detect EhAgo2-2 in JMP022-JMP027 strains. Anti-actin (MP Biomedicals) was used to detect the loading control.

### *S*. *cerevisiae* growth conditions, genetic manipulation and induction

All *S*. *cerevisiae* strains were generated in the W303-1B background and were grown at 30°C in YPD or SC solid or liquid media. Stable transformations were performed as in [56] with the following modifications: 100ml of cells were washed with 5ml of LiTE mix (100mM lithium acetate, 10mM Tris-HCl, 1mM EDTA, pH 7.5), resuspended in 1ml LiTE mix, and incubated at 30°C for 1hr. Transformation reaction (200μl cells, 10μl denatured salmon sperm DNA (Invitrogen), 5μg linearized DNA) was incubated at 30°C for 30 minutes followed by a 30 minute incubation at 30°C with 1ml of PEG mix (40% polyethylene glycol 33550 (Sigma), 100mM lithium acetate, 10mM Tris-HCl, 1mM EDTA, pH 7.5). Reaction was heat shocked at 42°C for 15 minutes and grown on selective media for 2–3 days at 30°C. To induce production of the silencing constructs, strains were grown to OD_600_ = 0.6 in selective media containing 1% galactose and 1% raffinose instead of 2% glucose. Samples from the same culture were used for RNA, protein, and flow cytometry analyses. Multiple transgenic clones per strain were examined.

### *S*. *cerevisiae* plasmid and strain construction

All *S*. *cerevisiae* plasmids used and generated in this study are listed in S4 Table. EhRNaseIII (EHI_068740) and EhAgo2-2 (EHI_125650) were codon-optimized for expression in *S*. *cerevisiae* by GenScript and incorporated the following restriction sites: EhRNaseIII (5’-XbaI, 3’-XhoI), EhAgo2-2 (5’-SpeI, 3’-XhoI). The ScaDcr1 sequence was removed from the parental plasmid (pRS405-P_TEF_-ScaDcr1) and codon-optimized EhRNaseIII was cloned in its place. A 3xMyc tag starting with ATG was cloned into the XbaI site to generate pRS405- P_TEF_-3xMyc-EhRNaseIII. To generate pRS405- P_TEF_-3xMyc-EhRNaseIII-dd, codon-optimized 3xMyc-EhRNaseIII (amino acids 1–133 of EhRNaseIII) was fused C-terminus of *S*. *castellii* Dcr1 (amino acids 265–610) and cloned into the pRS405-P_TEF_-ScaDcr1 plasmid in lieu of ScaDcr1. The ScaAgo1 sequence was removed from the pRS404- P_TEF_-ScaAgo1 plasmid and replaced by codon-optimized EhAgo2-2 to generate pRS404- P_TEF_-EhAgo2-2. To generate strains JMP019-021, pRS405- P_TEF_-3xMyc-EhRNaseIII was linearized with BstEII and integrated into DPB249-251, respectively. To generate strains JMP022-024, pRS404- P_TEF_-EhAgo2-2 was linearized with Bsu361 and integrated into JMP019-021, respectively. To generate strains JMP025-027, pRS404- P_TEF_-ScaAgo1 was linearized with HindIII and integrated into JMP019-021, respectively. To generate strains JMP028-030, pRS405- P_TEF_-3xMyc-EhRNaseIII-dd was linearized with BstEII and integrated into DPB249-251, respectively. To generate strains JMP031-034, pRS404- P_TEF_-ScaAgo1 was linearized with HindIII and integrated into JMP028-030, respectively. All strains used or generated in this study are listed in S5 Table.

### RNA isolation and Northern blot analysis

For *S*. *cerevisiae* samples, total RNA was isolated using the hot phenol method as described in [46]. High resolution Northern blot analysis was performed as described in [40) using 15μg of total RNA unless otherwise stated and probed using [γ-^32^P]dATP end-labeled antisense oligonucleotides (GFP siRNA- ACCATTATCAACAAAATACTCCAATTGGCGATGGCCCTGTCCTTTTACCA, U6 snRNA- TATGCAGGGGAACTGCTGAT). Small RNA-enriched material was isolated using the small RNA-enriched protocol of the miRVANA kit (Ambion) according to manufacturer’s instructions. Northern blots detecting mRNA were performed according to standard protocols [57] using 4μg of DNaseI-treated total RNA and probed using [α-^32^P] dATP internally labeled PCR products. The PCR probes were amplified from *S*. *cerevisiae* DPB249 genomic DNA using MasterPure Yeast DNA Purification Kit (Epicenter) and using the following primers: GFP (F- GTCAGTGGAGAGGGTGAAGG, R- TACATAACCTTCGGGCATGG), PYK1 (CDC19) (F- CACCACCGATGACAAGTACG, R- GCGGTTCTGATGAAAGAAGC). For statistical analysis where indicated, GFP mRNA band intensities for each strain were normalized against PYK1 in three independent experiments and the normalized value of JMP025 strain, where GFP is expressed and no silencing construct is present, was set to 1. Significance (p < 0.05) was determined by t-test. Blots were applied to a phosphorscreen and analyzed by Personal Molecular Imager (BioRad) and Quantity One (BioRad) where contrast was adjusted across the entire image.

### Flow cytometry analysis

Induced *S*. *cerevisiae* strains were washed with 1X TBS and resuspended in 1X TBS. Samples were analyzed for GFP fluorescence on FACScan (BD Biosciences) using the settings: FSC EØØ Amp = 2 Linear, SSC 350 Amp = 1 Linear, FL581 Log. A live cell gate was established using LIVE/DEAD FungaLight Yeast Viability Kit (Molecular Probes) and 10,000 cells were counted inside the live gate. Data were analyzed using FlowJo (TreeStar). Mean GFP intensity was calculated as in [44].

## Supporting Information

S1 Fig ClustalW alignment of RNaseIII proteins. Full-length sequences of EhRNaseIII (EHI_068740), *Aquifex aeolicus* RNaseIII (NP_213645), *Escherichia coli* RNaseIII (Accession AIL18413), *S*. *castellii* Dicer1 (Accession DAA12515), *Arabidopsis thaliana* Dicer (Accession AEZ02177), Human Dicer (Accession Q9UPY3), *Giardia lamblia* Dicer (Accession EDO77862) aligned using Geneious version R7 (Biomatters Ltd) (31). Alignment corresponding to the full-length sequence of EhRNaseIII shown. RNaseIII signature motif is shown in bold. Important catalytic residues are shown in red.
(PDF)

S2 Fig Amebic lysate degrades dsRNA *in vitro*. (Left) *In vitro* processing of radiolabeled of Eh-dsRNA by lysate from untransfected (UnTx) *E*. *histolytica* trophozoites. Reactions contained 3mM MgCl_2_ and 20mM potassium glutamate ± 20mM EDTA and were incubated for 2 hours at 37°C. 10μl of lysate were used in each reaction. One unit of recombinant *E*. *coli* RNaseIII (Ambion) was used as a positive control. dsRNA only reaction contained no protein and the buffer only reaction contained 10μl of transcription buffer (55). Extensive degradation was seen with amebic lysates but no specific cleavage products were detected. (Right) Small volumes of amebic lysate degrade dsRNA *in vitro*. Varying amounts of crude extract from *E*. *histolytica* trophozoites overexpressing Myc-EhRNaseIII (where 1X is 10μl) were incubated with radiolabeled Eh-dsRNA substrate for 2 hours at 37°C with 3mM MgCl_2_ and 20mM potassium glutamate ± 20mM EDTA. All reactions were supplemented with 40U of SUPERNase·In RNase Inhibitor (Ambion) and 30U of Protector RNase Inhibitor (Roche Diagnostics). The dsRNA only reaction contained no protein and the buffer only reaction contained 10μl of lysis buffer. Recombinant *E*. *coli* RNaseIII (Ambion) served as a positive control. Both positive and negative controls worked as expected.
(PDF)

S1 Table Final hits from bioinformatics analysis. Hits from bioinformatics analysis with e-values ≤ 0.5. The domain sought, the HMM used to search the database, the type of database searched, the Gene ID, the genome annotation, the raw score, and the e-value shown for each hit.
(PDF)

S2 Table RNaseIII domain sequences for multiple sequence alignment. RNaseIII domain sequences retrieved from Pfam database used to build RNaseIII clustalw alignment. UniProt entry ID, gene description, organism, and residues of RNaseIII domain in sequences shown.
(PDF)

S3 Table PAZ domain sequences for multiple sequence alignment. PAZ domain sequences retrieved from Pfam database used to build PAZ clustalw alignment. UniProt entry ID, gene description, organism, and residues of PAZ domain in sequences shown.
(PDF)

S4 Table *S*. *cerevisiae* plasmids used and generated in this study. (PDF)

S5 Table *S*. *cerevisiae* strains used and generated in this study. (PDF)
