# Supplementary material for: A Single RNaseIII Domain Protein from Entamoeba histolytica Has dsRNA Cleavage Activity and Can Help Mediate RNAi Gene Silencing in a Heterologous System
Source: PLoS One. 2015 Jul 31;10(7):e0133740. doi: 10.1371/journal.pone.0133740 (PMC4521922; doi:10.1371/journal.pone.0133740)
Supplement: S1 Fig — Full-length sequences of EhRNaseIII (EHI_068740), Aquifex aeolicus RNaseIII (NP_213645), Escherichia coli RNaseIII (Accession AIL18413), S. castellii Dicer1 (Accession DAA12515), Arabidopsis thaliana Dicer (Accession AEZ02177), Human Dicer (Accession Q9UPY3), Giardia lamblia Dicer (Accession EDO77862) aligned using Geneious version R7 (Biomatters Ltd) (31). Alignment corresponding to the full-length sequence of EhRNaseIII shown. RNaseIII signature motif is shown in bold. Important catalytic residues are shown in red. (PDF) [file pone.0133740.s001.pdf]

Figure S1

```
         EhRNaseIII   ----MSSTTLHNAMQYTAFDVLSSILN--------------------------------- 23
   A. aeolicus RIII   -------------MK--MLEQLEKKLG---------------------------YTFK-- 16
        E. coli RIII   -------------MNPIVINRLQRKLG---------------------------YTFN-- 18
S. castellii Dicer   DQ-LNTMAKHGNDLEKSIANSPAMSVASCLNQVRPTLDI---------KNIFDHYKFENN 102
 A. thaliana Dicer   EDLIGKIVTAAHSGKRFYVDSICYDMSAETSFPRKEGYLGPLEYNTYADYYKQKYGVDLN 1245
       Human Dicer   DLTAINGLSYNQNLANGSYDLANRDFCQGNQLNYYKQEIPVQP--TTSYSIQNLYSYENQ 1228
     Giardia Dicer   CLLLPDEFDLIRVQALQFLPEIAKHICDIQN---------------------TICALDKS 285

         EhRNaseIII   -----------LMKADPLYDLLQLNQAYS------------------------------- 41
   A. aeolicus RIII   --------------DKSLLEKALTHVSY-------------------------------- 30
        E. coli RIII   --------------HQELLQQALTHRSA-------------------------------- 32
S. castellii Dicer   SSPVDPYVHYPVCSDQNLENLAFIHRSLP------------------------------- 131
 A. thaliana Dicer   CKQQPLIKGRGVSYCKNLLSPRFEQSGESETVLDKTYYVFLPPELCVVHPLSGSLIRGAQ 1305
       Human Dicer   PQPSDECTLLSNKYLDGNANKSTSDGSPVMAVMPG----------------------TTD 1266
     Giardia Dicer   FPDCGRIGGERYFAITAGLRLDQGRGRGLAG----------------------------- 316

         EhRNaseIII   ----------------------------------------SQDQEYEKNEFYGDSYLEER 58
   A. aeolicus RIII   ----------------------------------------SKKEHYETLEFLGDALVNFF 50
        E. coli RIII   ----------------------------------------SSK-HNERLEFLGDSILSYV 51
S. castellii Dicer   ------------------------------NMNVKLTELQKTVMSNERLEFLGDSWLGAL 161
 A. thaliana Dicer   RLPSIMRRVESMLLAVQLKNLISYPIPTSKILEALTAASCQETFCYERAELLGDAYLKWV 1365
       Human Dicer   TIQVLKGRMDSEQSPSIGYSSRTLGPNPGLILQALTLSNASDGFNLERLEMLGDSFLKHA 1326
     Giardia Dicer   ------------------------------WRTPFGPFGVSHTDVFQRLELLGDAVLGFI 346

         EhRNaseIII   ASSLVLKFLRKYEQIPFEMYSGLRIHTVKNQTLGEIFDLLHLGDTKTFE----------- 110
   A. aeolicus RIII   IVDLLVQYSPNKREGFLSPLKAYLISEEFFNLLAQKLELHKFIRIKRG------------ 98
        E. coli RIII   IANALYHRFPRVDEGDMSRMRATLVRGNTLAELAREFELGECLRLGPG------------ 99
S. castellii Dicer   VAYIIYKKYPYANEGALSKMKEAIVNNNNLEKICEKLGFKERLKENIPR----------- 210
 A. thaliana Dicer   VSRFLFLKYPQKHEGQLTRMRQQMVSNMVLYQFALVKGLQSYIQADRFAPSRWSAPGVPP 1425
       Human Dicer   ITTYLFCTYPDAHEGRLSYMRSKKVSNCNLYRLGKKKGLPSRMVVSIFDPPVNWLPPG-- 1384
     Giardia Dicer   VTARLLCLFPDASVGTLVELKMELVRNEALNYLVQTLGLPQLAEFSNN------------ 394

         EhRNaseIII   --------------------------------------------------KKKKGDLVES 120
   A. aeolicus RIII   -----------------------------------------KIN------ETIIGDVFEA 111
        E. coli RIII   -----------------------------------------ELKSGGFRRESILADTVEA 118
S. castellii Dicer   -----------------------------------------SSMKIKDRLTKNYADCVEA 229
 A. thaliana Dicer   VFDEDTKDGGSSFFDEEQKPVSEENSDVFEDGEMEDGELEGDLSSYRVLSSKTLADVVEA 1485
       Human Dicer   ---YVVNQDKSNTDKWEKDEMTKDCMLANGKLDEDYEEEDEEEESLMWRAPKEEADYEDD 1441
     Giardia Dicer   ----------------------------------------------LVAKSKTWADMYEE 408

         EhRNaseIII   LIG--GCVLLSQRENATLFLLFAHALIDYIFYHSS------------------------- 153
   A. aeolicus RIII   LWA--AVYIDSGRDANFTRELFYKLFKEDILSAIK------------------------- 144
        E. coli RIII   LIG--GVFLDS--DIQTVEKLILNWYQT-RLDEIS------------------------- 148
S. castellii Dicer   YIG--ALVIDRFSTEFNDVALWLEELSEEHFIELG------------------------- 262
 A. thaliana Dicer   LIG--VYYVEGGKIAANHLMKWIGIHVEDDPDEVDGTLKNVNVPESVL-----------K 1532
       Human Dicer   FLEYDQEHIFIDNMLMGSGAFVKKISLSPFSTTDSAYEWKMPKKSSLGSMPFSSDFEDFD 1502
     Giardia Dicer   IVG--SIFTGPNGIYGCEEFLAKTLMSPEHSKTVG------------------------- 441

         EhRNaseIII   -------YIYFNANPPKLVKEEIITDIQNWFKDKLFYYRSSLEKYQTDPTNFYLEEQHVS 206
   A. aeolicus RIII   -------EGRVKKDYKTILQEITQKRWKERPEYRLISVEGPHHKKKFIVEAKIKEYRT-- 195
        E. coli RIII   -------PGDKQKDPKTRLQEYLQGRHLPLPTYLVVQVRGEAHDQEFTIHCQVSGLSEPV 201
S. castellii Dicer   -------PMMVKEPLNKNAKGELGAFLQFNNIGAKISYKRLNDKSPFKVEVRLGNNLLG- 314
 A. thaliana Dicer   SIDFVGLERALKYEFKEKGLLVEAITHASRPSSGVSCYQRLEFVGDAVLDHLITRHLFFT 1592
       Human Dicer   YSSWDAMCYLDPSKAVEEDDFVVGFWNPSEENCGVDTGKQSISYDLHTEQCIADKSIADC 1562
     Giardia Dicer   -----SACPDAVTKASKRVCMGEAGAHEFRSLVDYACEQGISVFCSSRVSTMFLERLRDI 496

         EhRNaseIII   VDLFDD---KEEVDTNDIDYILSSSDPLDFG---------------------IPEPSFNM 242
   A. aeolicus RIII   LGEGKS---KKEAEQRAAEELIKLLEESE------------------------------- 221
        E. coli RIII   VGTGSS---RRKAEQAAAEQALKKLELE-------------------------------- 226
S. castellii Dicer   IGDGSN---VREAEQRAAMEALAQPKLIQKYSLHDIELERGMIEEADNVPQLLKSAEIPN 371
 A. thaliana Dicer   YTSLPP---GRLTDLRAAAVNNENFARVAVKHKLHLYLRHGSSALEKQIREFVKEVQTES 1649
       Human Dicer   VEALLGCYLTSCGERAAQLFLCSLGLKVLPVIKRTDREKALCPTRENFNSQQKNLSVSCA 1622
     Giardia Dicer   PAEDMLDWYRLGIQFSHRSGLSGPGGVVSVIDIMTHLARGLWLGSPGFYVEQQTDKNESA 556

         EhRNaseIII   TTSPSWNSFVHPSQ---------------------------------------------- 256
   A. aeolicus RIII   ------------------------------------------------------------ 221
        E. coli RIII   ------------------------------------------------------------ 226
S. castellii Dicer   QTPQSHHSTEEIDEEEGLTFPHSNPFLKGTPPAVLPEIVTNQ-------------IPQAI 371
 A. thaliana Dicer   SKPGFNSFGLGDCKAPKVLGDIVESIAGAIFLDSGKDTTAAWK-------------VFQP 1696
       Human Dicer   AASVASSRSSVLKDSEYGCLKIPPRCMFDHPDADKTLNHLISGFENFEKKINYRFKNKAY 1682
     Giardia Dicer   CPPTIPVLYIYHR----------------------------------------------- 569
```
