# Supplementary figures and images for: A Single RNaseIII Domain Protein from Entamoeba histolytica Has dsRNA Cleavage Activity and Can Help Mediate RNAi Gene Silencing in a Heterologous System
Source: PLoS One. 2015 Jul 31;10(7):e0133740. doi: 10.1371/journal.pone.0133740 (PMC4521922; doi:10.1371/journal.pone.0133740)

**Figure S2**

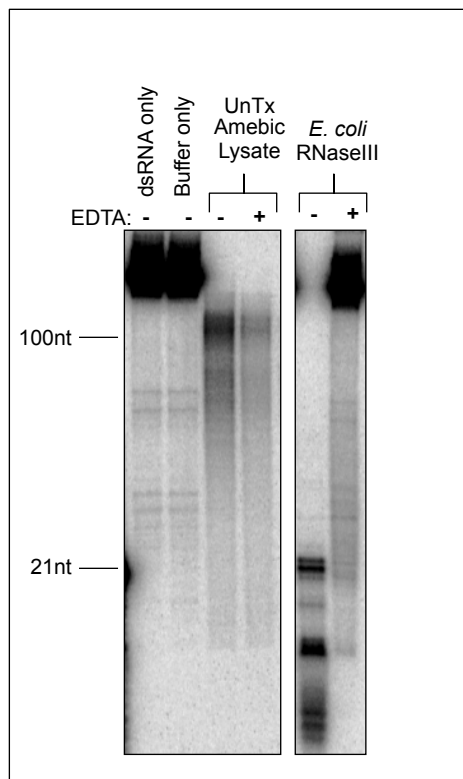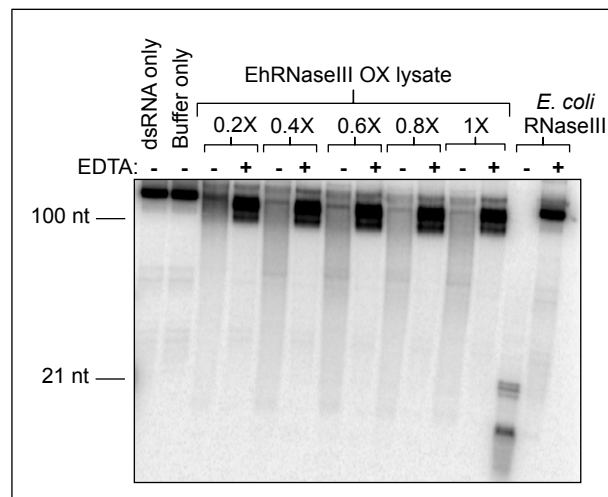

Supplement: S2 Fig — (Left) In vitro processing of radiolabeled of Eh-dsRNA by lysate from untransfected (UnTx) E. histolytica trophozoites. Reactions contained 3mM MgCl2 and 20mM potassium glutamate ± 20mM EDTA and were incubated for 2 hours at 37°C. 10μl of lysate were used in each reaction. One unit of recombinant E. coli RNaseIII (Ambion) was used as a positive control. dsRNA only reaction contained no protein and the buffer only reaction contained 10μl of transcription buffer (55). Extensive degradation was seen with amebic lysates but no specific cleavage products were detected. (Right) Small volumes of amebic lysate degrade dsRNA in vitro. Varying amounts of crude extract from E. histolytica trophozoites overexpressing Myc-EhRNaseIII (where 1X is 10μl) were incubated with radiolabeled Eh-dsRNA substrate for 2 hours at 37°C with 3mM MgCl2 and 20mM potassium glutamate ± 20mM EDTA. All reactions were supplemented with 40U of SUPERNase·In RNase Inhibitor (Ambion) and 30U of Protector RNase Inhibitor (Roche Diagnostics). The dsRNA only reaction contained no protein and the buffer only reaction contained 10μl of lysis buffer. Recombinant E. coli RNaseIII (Ambion) served as a positive control. Both positive and negative controls worked as expected. (PDF) [file pone.0133740.s002.pdf]
