# Supplementary material for: A Single RNaseIII Domain Protein from Entamoeba histolytica Has dsRNA Cleavage Activity and Can Help Mediate RNAi Gene Silencing in a Heterologous System
Source: PLoS One. 2015 Jul 31;10(7):e0133740. doi: 10.1371/journal.pone.0133740 (PMC4521922; doi:10.1371/journal.pone.0133740)
Supplement: S1 Table — Hits from bioinformatics analysis with e-values ≤ 0.5. The domain sought, the HMM used to search the database, the type of database searched, the Gene ID, the genome annotation, the raw score, and the e-value shown for each hit. (PDF) [file pone.0133740.s003.pdf]

S1 Table: Final hits from bioinformatics analysis

Domain	Hmm	Database	Gene ID	Genome Annotation	Score	e-value
RNaseIII	PF00636	Translated	EHI_068740	hypothetical protein	-4.8	0.011
RNaseIII	PF00636	Annotated	EHI_068740	hypothetical protein	-4.8	0.011
RNaseIII	RNaseIII_clustalw	Translated	EHI_068740	hypothetical protein	140.3	4.90E-38
RNaseIII	RNaseIII_clustalw	Annotated	EHI_068740	hypothetical protein	140.3	4.80E-39
PAZ	PAZ_clustalw	Translated	EHI_125650	PIWI, putative	73.4	6.70E-18
PAZ	PAZ_clustalw	Translated	EHI_186850	PIWI domain protein	151.7	1.90E-41
PAZ	PAZ_clustalw	Annotated	EHI_125650	PIWI, putative	151.7	1.80E-42
PAZ	PAZ_clustalw	Annotated	EHI_186850	PIWI domain protein	73.4	6.50E-19
PAZ	PF02170	Translated	EHI_125650	PIWI, putative	57.6	3.80E-13
PAZ	PF02170	Translated	EHI_186850	PIWI domain protein	89.8	8.10E-23
PAZ	PF02170	Annotated	EHI_125650	PIWI, putative	89.8	7.90E-24
PAZ	PF02170	Annotated	EHI_177170	hypothetical protein	-18.1	0.065
PAZ	PF02170	Annotated	EHI_186850	PIWI domain protein	57.6	3.70E-14
