# Supplementary material for: A Single RNaseIII Domain Protein from Entamoeba histolytica Has dsRNA Cleavage Activity and Can Help Mediate RNAi Gene Silencing in a Heterologous System
Source: PLoS One. 2015 Jul 31;10(7):e0133740. doi: 10.1371/journal.pone.0133740 (PMC4521922; doi:10.1371/journal.pone.0133740)
Supplement: S2 Table — RNaseIII domain sequences retrieved from Pfam database used to build RNaseIII clustalw alignment. UniProt entry ID, gene description, organism, and residues of RNaseIII domain in sequences shown. (PDF) [file pone.0133740.s004.pdf]

S2 Table: RNaseIII domain sequences from Pfam for mulitple sequence alignment

UniProt entry ID	Gene Description	Organism	Residues
A4VD87	Dicer-related RNase III protein Dcr2p	Tetrahymena thermophila	1443-1591
A4VD87	Dicer-related RNase III protein Dcr2p	Tetrahymena thermophila	1607-1720
Q6BCK1	Dicer-related RNase III protein Dcr1p	Tetrahymena thermophila	1224-1338
Q6BCJ9	Dicer-related RNase III protein Dcr2p	Tetrahymena thermophila	1623-1736
Q6BCJ9	Dicer-related RNase III protein Dcr2p	Tetrahymena thermophila	1328-1487
Q6BCJ7	Dicer-related RNase III protein Dcl1p	Tetrahymena thermophila	1048-1163
Q6BCJ7	Dicer-related RNase III protein Dcl1p	Tetrahymena thermophila	752-862
P34529	Endoribonuclease dcr-1	Caenorhabditis elegans	1348-1524
P34529	Endoribonuclease dcr-1	Caenorhabditis elegans	1614-1740
O01326	Protein drosha	Caenorhabditis elegans	870-957
O01326	Protein drosha	Caenorhabditis elegans	691-781
Q59FF9	Nuclear RNase III Drosha variant	Homo sapiens	417-430
A7E2D3	Endoribonuclease Dicer	Homo sapiens	1702-1824
A7E2D3	Endoribonuclease Dicer	Homo sapiens	1313-1575
Q9NRR4	Protein Drosha	Homo sapiens	1144-1233
Q9NRR4	Protein Drosha	Homo sapiens	966-1056
Q9UPY3	Endoribonuclease Dicer	Homo sapiens	1692-1814
Q9UPY3	Endoribonuclease Dicer	Homo sapiens	1303-1565
Q8CJ74	Ribonuclease III	Mus musculus	133-223
Q8CJ74	Ribonuclease III	Mus musculus	311-400
Q8R418	Endoribonuclease Dicer	Mus musculus	1686-1808
Q8R418	Endoribonuclease Dicer	Mus musculus	1303-1561
Q6ZQ23	Dicer1	Mus musculus	872-1130
Q6ZQ23	Dicer1	Mus musculus	1255-1377
Q5HZJ0	Ribonuclease III	Mus musculus	965-1055
Q5HZJ0	Ribonuclease III	Mus musculus	1143-1232
Q3UUV8	Dicer1	Mus musculus	294-416
Q6PAJ6	Rnasen protein	Mus musculus	823-913
Q6PAJ6	Rnasen protein	Mus musculus	1001-1090
Q6PF88	Rnasen protein	Mus musculus	712-801
Q6PF88	Rnasen protein	Mus musculus	534-624
Q80Z69	ribonuclease III-like protein	Mus musculus	791-880
Q80Z69	ribonuclease III-like protein	Mus musculus	613-703
Q27IU2	Dicer-1	Drosophila melanogaster	1685-1862
Q27IU2	Dicer-1	Drosophila melanogaster	1972-2043
Q2Q3V5	Dicer-2	Drosophila melanogaster	1462-1621
Q2Q3V5	Dicer-2	Drosophila melanogaster	1203-1375
Q27IU3	Dicer-1	Drosophila melanogaster	1972-2043
Q27IU3	Dicer-1	Drosophila melanogaster	1685-1862
A1ZAW0	Dicer-2	Drosophila melanogaster	1469-1628
A1ZAW0	Dicer-2	Drosophila melanogaster	1210-1382
Q2Q3W0	Dicer-2	Drosophila melanogaster	1203-1375
Q2Q3W0	Dicer-2	Drosophila melanogaster	1462-1621
Q27IU8	Dicer-1	Drosophila melanogaster	1685-1862
Q27IU8	Dicer-1	Drosophila melanogaster	1972-2043
Q2Q3V8	Dicer-2	Drosophila melanogaster	1462-1621
Q2Q3V8	Dicer-2	Drosophila melanogaster	1203-1375
Q9VCU9	Endoribonuclease Dcr-1	Drosophila melanogaster	2029-2150
Q9VCU9	Endoribonuclease Dcr-1	Drosophila melanogaster	1742-1919
Q27IU7	Dicer-1	Drosophila melanogaster	1972-2043
Q27IU7	Dicer-1	Drosophila melanogaster	1685-1862
Q6NP57	SD11113p	Drosophila melanogaster	1189-1361
Q6NP57	SD11113p	Drosophila melanogaster	1448-1607
Q7KNF1	Drosha	Drosophila melanogaster	833-923
Q7KNF1	Drosha	Drosophila melanogaster	1012-1098
Q95YG3	Double-strand-specific ribonuclease	Drosophila melanogaster	1210-1382
Q95YG3	Double-strand-specific ribonuclease	Drosophila melanogaster	1469-1628
Q2Q3V6	Dicer-2	Drosophila melanogaster	1203-1375
Q2Q3V6	Dicer-2	Drosophila melanogaster	1462-1621
Q27IU4	Dicer-1	Drosophila melanogaster	1685-1862
Q27IU4	Dicer-1	Drosophila melanogaster	1972-2043
Q960Y4	drosha	Drosophila melanogaster	756-842
Q960Y4	drosha	Drosophila melanogaster	577-667
Q152U6	Double-strand-specific Pac1 ribonuclease	Schizosaccharomyces pombe	172-262
Q09884	Dicer	Schizosaccharomyces pombe	930-1038
Q09884	Dicer	Schizosaccharomyces pombe	1120-1233
A8BQJ3	Endoribonuclease Dicer-like	Giardia intestinalis	333-418
A8BQJ	Endoribonuclease Dicer-like	Giardia intestinalis	646-734
O67082	Ribonuclease 3	Aquifex aeolicus	37-121
Q2EUJ9	Rnc	Escherichia coli	2-80
Q86QW6	Endoribonuclease Dicer-like	Giardia intestinalis	519-607
Q86QW6	Endoribonuclease Dicer-like	Giardia intestinalis	206-291
P0A7Y2	Ribonuclease 3	Escherichia coli	38-128
P0A7Y0	Ribonuclease 3	Escherichia coli (strain K12)	38-128
P0A7Y1	Ribonuclease 3	Escherichia coli O6	38-128
