# Supplementary material for: A Single RNaseIII Domain Protein from Entamoeba histolytica Has dsRNA Cleavage Activity and Can Help Mediate RNAi Gene Silencing in a Heterologous System
Source: PLoS One. 2015 Jul 31;10(7):e0133740. doi: 10.1371/journal.pone.0133740 (PMC4521922; doi:10.1371/journal.pone.0133740)
Supplement: S3 Table — PAZ domain sequences retrieved from Pfam database used to build PAZ clustalw alignment. UniProt entry ID, gene description, organism, and residues of PAZ domain in sequences shown. (PDF) [file pone.0133740.s005.pdf]

S3 Table: PAZ domain sequences from Pfam for mulitple sequence alignment

UniProt entry ID	Gene Description	Organism	Residues
Q389P5	Argonaute-like protein	Trypanosoma brucei brucei	289-422
Q6T6K0	Argonaute-like protein 1	Trypanosoma brucei	278-411
Q86NJ8	Protein PPW-1, isoform c	Caenorhabditis elegans	272-413
Q17567	Protein PRG-2	Caenorhabditis elegans	116-252
Q9TXN7	Protein WAGO-10	Caenorhabditis elegans	335-463
Q86B39	Protein ALG-2, isoform b	Caenorhabditis elegans	263-400
P91085	Protein WAGO-2	Caenorhabditis elegans	288-422
Q09249	Uncharacterized protein C16C10.3	Caenorhabditis elegans	391-504
O61931	Protein ERGO-1	Caenorhabditis elegans	421-557
Q3LTR7	Argonaute-like	Caenorhabditis elegans	385-522
Q27GU1	Protein CSR-1	Caenorhabditis elegans	215-358
Q9GZG4	Protein SAGO-2, isoform a	Caenorhabditis elegans	272-413
Q19598	Protein F19G12.3	Caenorhabditis elegans	3-73
Q9XVI3	Protein HPO-24	Caenorhabditis elegans	289-426
P90786	Protein PRG-1	Caenorhabditis elegans	218-354
O16386	Protein SAGO-1	Caenorhabditis elegans	274-413
Q9N3L7	Protein WAGO-11	Caenorhabditis elegans	319-439
Q86S33	Protein SAGO-2, isoform b	Caenorhabditis elegans	272-413
Q21770	Germ cell-expressed protein R06C7	Caenorhabditis elegans	321-455
P34529	Endoribonuclease dcr-1	Caenorhabditis elegans	785-961
Q21691	Protein NRDE-3	Caenorhabditis elegans	378-523
P34681	Putative protein tag-76	Caenorhabditis elegans	374-509
Q9XUY9	Protein F55C9.3	Caenorhabditis elegans	21-151
O16720	Protein ALG-2, isoform a	Caenorhabditis elegans	282-419
O62275	Protein WAGO-4;	Caenorhabditis elegans	317-451
Q9N585	Protein PPW-2	Caenorhabditis elegans	328-461
Q9HCK5	Protein argonaute-4	Homo sapiens	225-361
Q9UL18	Protein argonaute-1	Homo sapiens	233-369
Q9UPY3	Endoribonuclease Dicer	Homo sapiens	881-1055
Q96J94	Piwi-like protein 1	Homo sapiens	277-414
A4FVC0	EIF2C2 protein	Homo sapiens	184-320
Q5TA58	Protein argonaute-1	Homo sapiens	158-294
Q7Z3Z3	Piwi-like protein 3	Homo sapiens	292-429
Q9H9G7	Argonaute3;	Homo sapiens	236-372
Q9UKV8	Argonaute2	Homo sapiens	227-363
Q7Z3Z4	Piwi-like protein 4	Homo sapiens	271-407
Q8CGT8	Argonaute 4 protein	Mus musculus	441-485
Q8CJF8	Protein argonaute-4	Mus musculus	225-361
Q6ZQ23	Dicer1	Mus musculus	450-624
Q8CJF9	Argonaute3	Mus musculus	236-372
Q8CJG0	Argonaute2	Mus musculus	236-372
Q8R3Q7	Eif2c2	Mus musculus	1-42
Q8CJG1	Argonaute1	Mus musculus	233-369
A2VDG1	Eif2c1	Mus musculus	231-367
Q8CGT6	Piwi-like protein 4	Mus musculus	250-386
Q6P239	Eif2c2	Mus musculus	1-132
Q8BJP2	Eif2c1	Drosophila melanogaster	82-218
Q8CGU0	Eif2c1	Mus musculus	245-381
Q8R418	Endoribonuclease Dicer	Mus musculus	881-1055
Q7M738	Argonaute 3	Mus musculus	227-363
Q8CGT7	Argonaute 5 protein;	Mus musculus	191-282
Q3TBP7	Argonaute3	Mus musculus	236-372
Q8CDG1	Piwi-like protein 2	Mus musculus	386-523
Q9JMB7	Piwi-like protein 1	Mus musculus	278-415
Q8CGT9	Argonaute 2 protein	Mus musculus	244-356
Q2Q3V5	Dicer-2	Drosophila melanogaster	836-996
Q2Q3Y4	Protein argonaute-2	Drosophila melanogaster	227-365
Q7KY08	Argonaute protein	Drosophila melanogaster	310-446
Q27IU9	Dicer-1	Drosophila melanogaster	1039-1212
Q2Q3W0	Dicer-2	Drosophila melanogaster	836-996
Q2Q3Y1	Protein argonaute-2	Drosophila melanogaster	227-365
Q9VCU9	Endoribonuclease Dcr-1	Drosophila melanogaster	1096-1269
Q6NP57	SD11113p	Drosophila melanogaster	822-982
Q7PLK0	Argonaute 3, isoform D	Drosophila melanogaster	290-425
Q6NNZ4	piwi	Drosophila melanogaster	276-409
A4GND8	Argonaute3	Drosophila melanogaster	290-425
Q2Q3V4	Dicer-2	Drosophila melanogaster	836-996
Q32KD4	Argonaute-1, isoform A	Drosophila melanogaster	344-480
Q2Q3W1	Dicer-2	Drosophila melanogaster	836-996
Q960T1	AGO1	Drosophila melanogaster	2-97
Q9VKM1	piwi	Drosophila melanogaster	262-395
A7YFW6	Ago3	Drosophila melanogaster	23-158
Q27IR0	AGO1	Drosophila melanogaster	257-393
A8DYZ0	Aubergine, isoform C	Drosophila melanogaster	203-342
A4GUJ7	Ago3	Drosophila melanogaster	290-425
Q2Q3Y0	Protein argonaute-2	Drosophila melanogaster	227-365
Q9VUQ5	Protein argonaute-2	Drosophila melanogaster	602-740
Q2Q3Y3	Protein argonaute-2	Drosophila melanogaster	227-365
Q95YG3	Double-strand-specific ribonuclease	Drosophila melanogaster	843-1003
Q2Q3V6	Dicer-2	Drosophila melanogaster	836-996
Q6NP34	piwi	Drosophila melanogaster	290-423
O76922	AUBERGINE	Drosophila melanogaster	274-413
Q2Q3V7	Dicer-2	Drosophila melanogaster	836-996
Q27IU4	Dicer-1	Drosophila melanogaster	1039-1212
Q29AU1	GA18437	Drosophila pseudoobscura	1114-1287
Q28ZU1	GA19767	Drosophila pseudoobscura	345-481
Q29P29	GA19382	Drosophila pseudoobscura	191-330
Q29CN1	GA19370	Drosophila pseudoobscura	233-366
O74957	argonaute	Schizosaccharomyces pombe	212-349
A8BQJ3	Endoribonuclease Dicer-like	Giardia intestinalis	169-251
Q86QW6	Endoribonuclease Dicer-like	Giardia intestinalis	42-124
