# Supplementary material for: A Single RNaseIII Domain Protein from Entamoeba histolytica Has dsRNA Cleavage Activity and Can Help Mediate RNAi Gene Silencing in a Heterologous System
Source: PLoS One. 2015 Jul 31;10(7):e0133740. doi: 10.1371/journal.pone.0133740 (PMC4521922; doi:10.1371/journal.pone.0133740)
Supplement: S4 Table — (PDF) [file pone.0133740.s006.pdf]

S4 Table: *S. cerevisiae* plasmids used and generated in this study.

Plasmid	Description	Reference
pRS404-P_{TEF}-ScaAgo1	*S. cerevisiae* integration plasmid; *S. castellii* Argonaute1 under TEF promoter	20
pRS404-P_{TEF}-EhAgo2-2	*S. cerevisiae* integration plasmid; codon-optimized *E. histolytica* Argonaute2-2 for expression in *S. cerevisiae* under TEF promoter	This study
pRS405-P_{TEF}-ScaDcr1	*S. cerevisiae* integration plasmid; *S. castellii* Dicer1 under TEF promoter	20
pRS405-P_{TEF}-3xMyc-EhRNaseIII	*S. cerevisiae* integration plasmid; codon-optimized *E. histolytica* RNaseIII for expression in *S. cerevisiae* with N-terminal 3x-Myc tag under TEF promoter	This study
pRS405-P_{TEF}-3xMyc-EhRNaseIII-dd	*S. cerevisiae* integration plasmid; fusion of codon-optimized 3xMyc-*E. histolytica* RNaseIII (aa 1-133) and C-terminus of *S. castellii* Dicer1 (aa 265-610) for expression in under TEF promoter	This study
