# Supplementary material for: A Single RNaseIII Domain Protein from Entamoeba histolytica Has dsRNA Cleavage Activity and Can Help Mediate RNAi Gene Silencing in a Heterologous System
Source: PLoS One. 2015 Jul 31;10(7):e0133740. doi: 10.1371/journal.pone.0133740 (PMC4521922; doi:10.1371/journal.pone.0133740)
Supplement: S5 Table — (PDF) [file pone.0133740.s007.pdf]

S5 Table: *S. cerevisiae* Strains used and generated in this study.

Strain	Genotype	Species	Reference
DPB249	Mat α leu2-3,112 trp1-1 can1-100 ura3::EGFP(S65T)-KanMX6 ade2-1 his3-11, 15	*S. cerevisiae* W303-1B	20
DPB250	Mat α leu2-3,112 trp1-1 can1-100 ura3::EGFP(S65T)-KanMX6 ade2-1 HIS3::pGAL1-weakSC-GFP	*S. cerevisiae* W303-1B	20
DPB251	Mat α leu2-3,112 trp1-1 can1-100 ura3::EGFP(S65T)-KanMX6 ade2-1 HIS3::pGAL1-strongSC-GFP	*S. cerevisiae* W303-1B	20
DPB258	Mat α LEU2::pTEF-ScaDcr1 TRP1::pTEF-ScaAgo1 can1-100 ura3::EGFP(S65T)-KanMX6 ade2-1 his3-11, 15	*S. cerevisiae* W303-1B	20
DPB259	Mat α LEU2::pTEF-ScaDcr1 TRP1::pTEF-ScaAgo1 can1-100 ura3::EGFP(S65T)-KanMX6 ade2-1 HIS3::pGAL1-weakSC-GFP	*S. cerevisiae* W303-1B	20
DPB260	Mat α LEU2::pTEF-ScaDcr1 TRP1::pTEF-ScaAgo1 can1-100 ura3::EGFP(S65T)-KanMX6 ade2-1 HIS3::pGAL1-strongSC-GFP	*S. cerevisiae* W303-1B	20
DPB271	Mat α leu2-3,112 trp1-1 can1-100 ade2-1 his3-11, 15	*S. cerevisiae* W303-1B	20
JMP019	Mat α LEU2::pTEF-3xMyc-EhRNaseIII trp1-1 can1-100 ura3::EGFP(S65T)-KanMX6 ade2-1 his3-11, 15	*S. cerevisiae* W303-1B	This study
JMP020	Mat α LEU2::pTEF-3xMyc-EhRNaseIII trp1-1 can1-100 ura3::EGFP(S65T)-KanMX6 ade2-1 HIS3::pGAL1-weakSC-GFP	*S. cerevisiae* W303-1B	This study
JMP021	Mat α LEU2::pTEF-3xMyc-EhRNaseIII trp1-1 can1-100 ura3::EGFP(S65T)-KanMX6 ade2-1 HIS3::pGAL1-strongSC-GFP	*S. cerevisiae* W303-1B	This study
JMP022	Mat α LEU2::pTEF-3xMyc-EhRNaseIII TRP1::pTEF-EhAgo2-2 can1-100 ura3::EGFP(S65T)-KanMX6 ade2-1 his3-11, 15	*S. cerevisiae* W303-1B	This study
JMP023	Mat α LEU2::pTEF-3xMyc-EhRNaseIII TRP1::pTEF-EhAgo2-2 can1-100 ura3::EGFP(S65T)-KanMX6 ade2-1 HIS3::pGAL1-weakSC-GFP	*S. cerevisiae* W303-1B	This study
JMP024	Mat α LEU2::pTEF-3xMyc-EhRNaseIII TRP1::pTEF-EhAgo2-2 can1-100 ura3::EGFP(S65T)-KanMX6 ade2-1 HIS3::pGAL1-strongSC-GFP	*S. cerevisiae* W303-1B	This study
JMP025	Mat α LEU2::pTEF-3xMyc-EhRNaseIII TRP1::pTEF-ScaAgo1 can1-100 ura3::EGFP(S65T)-KanMX6 ade2-1 his3-11, 15	*S. cerevisiae* W303-1B	This study
JMP026	Mat α LEU2::pTEF-3xMyc-EhRNaseIII TRP1::pTEF-ScaAgo1 can1-100 ura3::EGFP(S65T)-KanMX6 ade2-1 HIS3::pGAL1-weakSC-GFP	*S. cerevisiae* W303-1B	This study
JMP027	Mat α LEU2::pTEF-3xMyc-EhRNaseIII TRP1::pTEF-ScaAgo1 can1-100 ura3::EGFP(S65T)-KanMX6 ade2-1 HIS3::pGAL1-strongSC-GFP	*S. cerevisiae* W303-1B	This study
JMP028	Mat α LEU2::pTEF-3xMyc-EhRNaseIII-dd trp1-1 can1-100 ura3::EGFP(S65T)-KanMX6 ade2-1 his3-11, 15	*S. cerevisiae* W303-1B	This study
JMP029	Mat α LEU2::pTEF-3xMyc-EhRNaseIII-dd trp1-1 can1-100 ura3::EGFP(S65T)-KanMX6 ade2-1 HIS3::pGAL1-weakSC-GFP	*S. cerevisiae* W303-1B	This study
JMP030	Mat α LEU2::pTEF-3xMyc-EhRNaseIII-dd trp1-1 can1-100 ura3::EGFP(S65T)-KanMX6 ade2-1 HIS3::pGAL1-strongSC-GFP	*S. cerevisiae* W303-1B	This study
JMP031	Mat α LEU2::pTEF-3xMyc-EhRNaseIII-dd TRP1::pTEF-Ago1 can1-100 ura3::EGFP(S65T)-KanMX6 ade2-1 his3-11, 15	*S. cerevisiae* W303-1B	This study
JMP032	Mat α LEU2::pTEF-3xMyc-EhRNaseIII-dd TRP1::pTEF-Ago1 can1-100 ura3::EGFP(S65T)-KanMX6 ade2-1 HIS3::pGAL1-weakSC-GFP	*S. cerevisiae* W303-1B	This study
JMP033	Mat α LEU2::pTEF-3xMyc-EhRNaseIII-dd TRP1::pTEF-Ago1 can1-100 ura3::EGFP(S65T)-KanMX6 ade2-1 HIS3::pGAL1-strongSC-GFP	*S. cerevisiae* W303-1B	This study
